# Interaction of contextual, setting and implementation factors on a podoconiosis intervention in Rural Ethiopia: Results from a qualitative study

**DOI:** 10.1371/journal.pone.0328237

**Published:** 2025-07-11

**Authors:** Kibur Engdawork, Getnet Tadele, Gail Davey, Papreen Nahar, Shahaduz Zaman

**Affiliations:** 1 College of Social Sciences, Addis Ababa University, Addis Ababa, Ethiopia; 2 Centre for Equitable Global Health Research, Brighton and Sussex Medical School, Brighton and Hove, England; 3 School of Public Health, Addis Ababa University, Addis Ababa, Ethiopia; Caleb University, NIGERIA

## Abstract

Social science perspectives enable more holistic approaches to the evaluation of health interventions. Balance between context, setting and implementation is key to the sustainable impact of health interventions. However, little is known about how the implementation of interventions against Neglected Tropical Diseases interacts with context and settings, or the influence of the interaction on intervention outcomes. We conducted a qualitative evaluation of a podoconiosis intervention in Northwestern Ethiopia, and collected data on intervention implementation, context, setting and relations among these elements between 10/04/2022 and 29/07/2022. Our system and network analyses revealed that the context, setting and implementation of the intervention interacted formally and informally across macro, meso and micro levels during intervention delivery and created constraints on the intervention. In future, implementers must design programs that can withstand the constraining effects of contextual and related factors to effectively deliver activities and institutionalize NTD services at the community level. These programs must be supported by simultaneous efforts to mobilize institutions and local actors to ensure reflection on priorities and local solutions. Integrating social science perspectives into intervention evaluation enables better analysis and understanding of the factors influencing health interventions.

## Introduction

Neglected Tropical Diseases (NTDs) are a group of tropical conditions that are caused by a variety of communicable and non-communicable pathogens. NTDs are highly prevalent in Africa and Asia and cause devastating health, social and economic consequences among more than 1 billion people. Interventions have been implemented to control NTDs through mass drug administration (MDA), water, sanitation, and hygiene (WASH), vector control, health education, and micronutrient supplementation [1].

Ethiopia shoulders the burden of several NTDs including podoconiosis, a condition that is caused by chronic exposure to volcanic red clay soil via walking barefoot [2]. Podoconiosis causes severe swelling of the lower legs of patients [2]. Ethiopia bears 45% of the total burden of podoconiosis with 1.5 million affected individuals [3]. The country also has about 35 million people at risk [4]. The government of Ethiopia has prioritized prevention and control of podoconiosis in its national NTD master plans [5].

In Ethiopia, some Non-Governmental Organizations (NGOs) have disseminated information about the disease to influence public discourse, enhance preventive action and reduce the stigma of patients. They also provide lymphedema services and support economic empowerment of patients [6]. However, little evidence exists about the nature of interventions against podoconiosis, their outcomes and factors that influence the implementation and outcome of the interventions.

Advances in evaluation have questioned traditional evaluation approaches that focus solely on the implementation process for failing to identify all the important factors behind the success or failure of interventions. This has resulted in the development of innovative evaluation frameworks that assess interventions in a more comprehensive manner [7–9].

Among these evaluation frameworks, the Context and Implementation of Complex Interventions (CICI) framework demonstrates how context, implementation and setting interact with one another to influence interventions [9]. Context refers to a set of active factors in which an intervention is embedded [10]. Context comprises seven domains: geographical, epidemiological, socio-cultural, socioeconomic, ethical, legal and political [9]. Implementation is an actively planned and deliberately initiated effort with the intention of bringing a given intervention into practice within a particular setting. Setting refers to the specific physical and organizational environment in which the intervention is put into practice [9].

The CICI framework considers interventions as events within a complex system and clarifies the interaction between context, setting and implementation when examining implementation success or failure [9]. Implementers should have a clear understanding of the nature of the interaction between these elements. These may be manifested through structures, institutions and agents in the systems in which the intervention takes place. Although there is no unifying conceptualization of structures and institutions, they are considered non-agential phenomena [11]. Structures can be understood as constraining and self-perpetuating forces of society that create patterns of behaviour and outcomes [12]. Structural mechanisms include factors that generate stratification and social class division in society and that define individuals’ socioeconomic positions. On the other hand, institutions are systems of established rules, conventions, norms, values and customs [11]. Agency can be understood as individuals’ capacity for free will and their ability to make health-related or other meaningful decisions [13].

To date, there has been little information about how contextual factors interact with settings and implementation of interventions and influence intervention outcomes. Indeed, studying interventions in this manner is a new to Africa and only a few studies have assessed the effect of contextual factors on health interventions in Sub-Saharan Africa [14]. We evaluated a health intervention known as “Next steps for podoconiosis patients in Amhara region” that was being jointly implemented by two NGOs known as International Orthodox Christian Charities (IOCC) and National Podoconiosis Action Network (NaPAN). Informed by the CICI framework, the study aimed to examine how the context, the implementation and the setting of the NGOs interacted with one another to affect intervention outcomes. Studying these factors is important to identify macro-level effect modifiers such as structures and institutions at national and regional environment, meso-level factors, i.e., community organizations and resources, and micro-level factors such as agents who influence the impact of an intervention either in positive or negative ways [7].

## Materials and methods

### Study setting

The two NGOs implemented the intervention in seven districts of Amhara region in Northwestern Ethiopia. We purposefully selected Yilmana Densa district from West Gojjam and Dera District from South Gondar as they had a 10% podoconiosis prevalence rate. There were 6,400 households in these districts and we purposefully selected four *kebeles* (smallest administrative units); namely, Shime from Dera District, and Agita, Fetlo and Abika from Yilmana Densa District. During the intervention, 314 affected individuals were living in these *kebeles.* Shime *kebele* had the highest burden of podoconiosis in Dera District with 145 registered patients, while Agita, Fetlo and Abika ke*beles* were also relatively high-burden kebeles with 62, 56 and 51 affected individuals, respectively. We conducted observations at the offices of both NGOs in Addis Ababa, Ethiopia, where they are headquartered.

### Study design

We employed a qualitative study design. We conducted focus group discussions, observation, key informant interviews, and IDIs between April and August 2022 to learn about the context, the setting and implementation process and the relationship between these. The lead author and research assistant (who holds a Master’s degree in social science) collected the data. The social network perspective views characteristics of the actors as arising out of the structural or relational process [15]. Hence, the collection of relational data is critical for the analysis of social networks. The CICI framework and the document analysis informed the development of interview, discussion and observation guides. The set of issues in the checklist were revised during the interview sessions.

The study team first analysed the “Next Steps for Podoconiosis Patients in Amhara Region, Ethiopia, project document [16] to learn about the implementation aspect of the intervention, i.e., the goal, objectives, agents, strategies, processes etc. We further analysed government documents to explore the political contexts, i.e., national programs that may have affected the implementation of the intervention.

Subsequently, we purposefully selected 32 individuals for in-depth interviews. Fourteen of the participants were females. Twenty-eight patients had no formal education, and they engaged in agricultural activities. We engaged participants who were living in the study areas permanently and were willing to take part in the study. The participants were between the age of 18 and 64 and were physically and mentally able to complete a 45-to-60-minute in-depth interview. To capture maximum variation, the study involved different age categories, both genders and community members with various levels of education and economic status. The interview topics helped explore the lives of affected individuals, their interaction with health professionals and involvement in social activities and the health intervention. We conducted interviews privately in individuals’ compounds.

We spent 17 days over an extended period in July 2022 at the NGOs’ offices to observe the physical spaces, documents, regular activities, staff and their workplace interaction. We also observed health education at healthcare facilities and social interaction among community members at social gatherings in the four kebeles of the two rural districts. The observation was conducted to identify contextual factors such as epidemiological, socio-economic context and the setting that influenced the intervention.

We held key informant interviews with 20 informants. We interviewed five staff of the implementing NGOs to assess the major health issues addressed by the program, their interaction with government offices, other stakeholders and the targeted community during the course of intervention delivery. We also interviewed one government official, 11 local health professionals and three local administrators to examine existing interactions and collaborations.

In parallel, we conducted four FGDs with unaffected community representatives to collect relational data: two with women participants in each district. Six men participated in the FGD at Yilmana Densa district; and eight individuals took part in the remaining three FGDs. We selected participants who permanently lived in the communities and were identified as community leaders by the local administrators, patients and health professionals. Participants were invited to discuss the relationships among themselves and with community organizations and health care providers, focusing on their health and social needs. We conducted the discussions at healthcare facilities on Sundays. Table 1 summarises the locations and participants for each study type.

**Table 1:** Summary of study setting, methods and participants.

Region	District	*Kebele*	IDI	FGD	KII	Observation
Amhara Regional State	Dera	Shime	8	2 (14 participants)	5	1
	Yilmana Densa	Agita	10	2 (16 participants)	4	1
		Abika	6		2	1
		Fetlo	8		2	1
	Bahirdar				2	
Addis Ababa	–	–			5	2
Total			32	4 (30 participants)	20	5

### Data analysis

We analysed the qualitative data using NVivo version 12 data analysis software. We recorded in depth interviews and key informant interviews in the Amharic language and transcribed and translated interviews into English. The lead author imported the transcripts, observation notes and intervention document into NVivo software and coded the data line by line considering interaction of the context, setting and implementation (See Supplementary Material, S1 Data). Research team members evaluated the codes, resolved some inconsistencies, and agreed on the final version of coding and themes. We used quotations as evidence to substantiate our themes and arguments.

Examining the complex interactions between the context, setting and implementation can be hard [9]. As suggested by exponents of the CICI framework, we developed a system-based logic model to visualize the relationship between context, implementation and setting [9,17]. We included relationship direction and type of effect (See Supplementary Material, S2 Data). A system logic model was developed to depict the relationship between the context, implementation and setting. The qualitative data were quantitatively analysed to examine the interactions of actors representing government, NGOs and community associations with intervention targets using a social network analysis. The analysis emphasized the impact of the relations of “actors”. “Actors” can be individual persons or social entities such as organizations [18]. A “network” is defined as a collection of actors connected to one another [19]. We considered the agents, i.e., implementers and targets of the intervention along with the major collaborators to be actors. These included the Ethiopian Ministry of Health, IOCC/NaPAN, the regional health bureau, district administration, health professionals at healthcare facilities, health extension workers (HEWs), traditional healers, schools, religious leaders, community leaders, traditional associations, Health Development Army (HDA) leaders—a network of women volunteers organized to promote health, family members and affected individuals. The elements of actors were classified as label, type, and description in an Excel spreadsheet. We indicated connections between actors along with their type, direction, i.e., mutual or direct and description of relationship. The data were entered into an Excel spreadsheet, which was consolidated and imported to Kumu mapping software (Kumu) for analysis and generation of stakeholder maps (See Supplementary Material, Data 3). We studied the links between these actors using three network measures: degree centrality, betweenness centrality and closeness centrality.

Degree centrality of node x is calculated using the following equation:


$$\text{degCentrality}(\text{x})=\text{deg}(\text{x})/({\text{Nodes}}_{\text{Total}}-1))/({\text{Nodes}}_{\text{Total}}-2).$$

where:

- Nodes_Total_ = The number of nodes in the network
- deg(x) = The number of nodes connected to node x [15,20].

Betweenness centrality of node x is calculated using the following equation:


$$\text{btwCentrality}(\text{x})=\Sigma_{\text{a,b}\in \text{Nodes}}({\text{paths}}_{\text{a,b}}(\text{x})/{\text{paths}}_{\text{a,b}})$$

where:

- Nodes = All the nodes in the network
- paths_a,b_ = The number of shortest paths between all nodes a and b
- paths_a,b_(x) = The number of shortest paths between nodes a and b that connect through node [20]

Closeness centrality of node x is calculated using the following equation:


$$\text{closeCentrality}(\text{x})=(\text{nodes}(\text{x,y})/({\text{Nodes}}_{\text{Total}}-1))*(\text{nodes}(\text{x,y})/\text{dist}(\text{x,y}{)}_{\text{Total}})$$

where:

- Nodes_Total_ = The number of nodes in the network
- nodes(x,y) = The number of nodes that are connected to node x
- dist(x,y)_Total_ = The sum of the shortest path distances from node x to other nodes [20].

The results of each measure are presented in Table 2 and Fig 3 to depict the relationship between the actors.

**Table 2:** Scores of degree centrality, closeness centrality and betweenness centrality.

Actors	Degree centrality	Closeness centrality	Betweenness centrality
Patients	0.038	0.395	0.134
HEWs	0.033	0.573	0.335
Health professionals	0.028	0.531	0.371
Traditional Associations	0.028	0.395	0.029
Family members	0.028	0.329	0.054
Churches	0.023	0.395	0.028
Community leaders	0.023	0.437	0.263
IOCC/NaPAN	0.019	0.448	0.036
Religious leaders	0.019	0.426	0
Regional health bureaus	0.014	0.417	0.093
District Administration	0.009	0.385	0.024
Schools	0.009	0.254	0
Ministry of Health	0.009	0.328	0
Traditional healers	0.004	0.298	0
Patient associations	0.004	0	0
HDA	0	0	0

**Fig 1:**
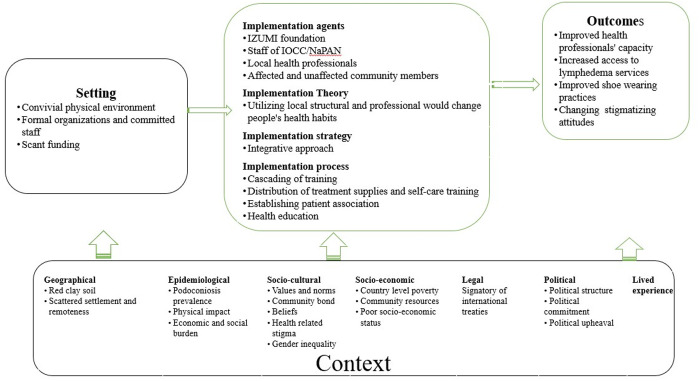
System-based logic model depicting the relationship between context, implementation and setting.

**Fig 2:**
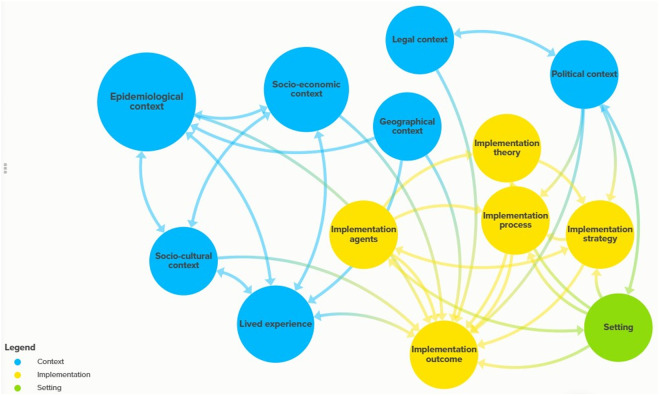
Complex system map of the context, implementation and the setting.

**Fig 3:**
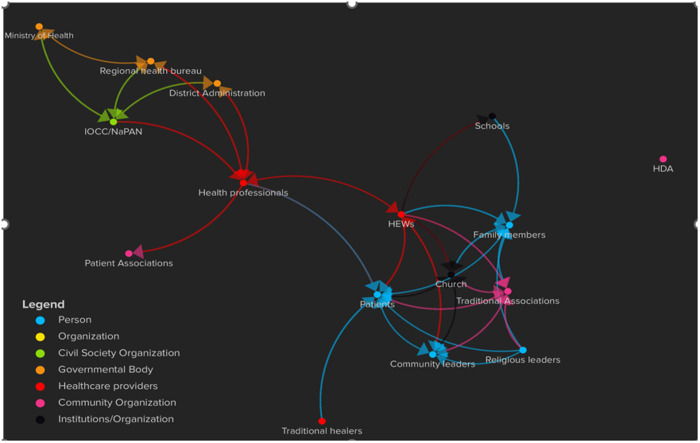
Graph of interactions between major actors.

### Ethical consideration

The Research Governance and Ethics Committee (RGEC) of Brighton and Sussex Medical School (BSMS) (Reference: ER/BSMS9E3G/8) and the Ethiopian Society of Sociologists, Social Workers and Anthropologists (ESSSWA) (Reference: ESSSWA 019/21) granted ethical clearance for the study. We were given permission to conduct the study by the Amhara Public Health Institute, regional and zonal health bureaus.

We used an easily understandable information sheet. Participants voluntarily took part in the study after having a clear understanding of the aims of the study. Written informed consent was obtained from the participants. We read out consent forms for each participant and they confirmed their voluntary participation in the study by signing on consent forms. Participants who cannot read and write confirmed voluntary participation by thumbprints. Participation in the study was confidential. Personal identification data were not collected by the study and study participants were anonymized using interview numbers and questionnaire ID numbers. Using our prior fieldwork experience, we provided 200 ETB ($4) to In-depth interview and FGD participant to compensate for their time.

### Validity, reliability and positionality

Interviews were audio-recorded, and the lead author and his research assistant made field notes during observation, discussions and interviews. After the data were collected, we summarized the main points and read them out to participants for confirmation. Most respondents confirmed the data while some of the respondents provided additional information. The data were transcribed into Amharic, translated into English and the lead author coded the transcripts using qualitative data coding techniques. He then developed a codebook that consists of the CICI constructs, themes, explanations and supporting quotes (See Supplementary Information 1). All other authors, who have extensive experience in qualitative research, reviewed and commented on the codes and themes.

After the completion of interviews, we summarized the main points and read them out to our interviewees for confirmation. We acknowledge that our identity could influence the research process and have made attempts to reduce its impact, or at least to be aware of our biases. We used to spend the whole day in the study area to better understand the setting and our participants. We used to sit in the same physical space as the study participants. Even when we are not interviewing individuals, the lead author and a research assistant used to roam around and take part in social and religious activities. We attended a couple of funeral ceremonies. We used to sit and listen to small talk and personal stories of affected individuals if the informal interaction would make us understand their lives better.

## Results

### Interaction of the context, setting and implementation of the intervention

The study has identified the implementation process of a health intervention against the NTD podoconiosis in rural Ethiopia and its outcomes. We also identified five contextual domains of the CICI framework that affected the implemented intervention. These included geographical, epidemiological, sociocultural socioeconomic, socio-economic, legal, political contexts. Further, the patients’ lived experience was among the important factors that affected the involvement and outcome of the intervention. The study indicates the physical and environmental setting of the intervention implementing NGOs also interacted with the implementation and contextual factors to influence outcomes.

Staff of the NGOs said that they utilized government healthcare facilities in a bid to mainstream podoconiosis services to local healthcare structures. The intervention provided training for health professionals and the public and distributed supplies to affected individuals over a three-month period. The staff of the NGOs reported that they reached hundreds of patients in the four *kebeles*. Our prior quantitative analysis of the same intervention revealed that 26 out of 42 patients reported being involved in the intervention. Most of the IDI participants also stated that they have received health education, washing basin, Vaseline and shoes from the intervention implementers.

The intervention implementers informed the study that they intend to enhance the capacity of local health professionals to provide lymphedema services. Health professionals reported that they have received training on podoconiosis and the training improved their capacity to provide treatment for patients. However, some of the project activities were not implemented as intended. For instance, not all HEWs received training from health professionals. One HEW said, “We have not received any training on podoconiosis. It was given only to the health staff at the health care facilities, not for health extension workers. We were given just a quick briefing or orientation by the health staff and observed the demonstrations performed by the health staff on how to wash and bandage the affected part of the feet. That was not sufficient for us to understand the condition in detail (KII, HEW at Dera District) Health Development Army leaders did not take part in the intervention as the network was not functional during the intervention.

Due to lack of follow up, a few patients defaulted from the project activities. An excerpt from one patient reads, “I went to the healthcare facility and health professionals showed us how to take care of our feet and provide us with soaps. I didn’t go in the second round. Why would I travel with my ill leg for three bars of soap”? (IDI, a 45-year-old affected man). Another excerpt reads as follows, “The health professional told me that it is curable, but I don’t believe it is true. There is no promising improvement in my condition since I have started the treatments. I gave up hope all together. So, I stopped going to the health care facility and I don’t follow the instructions anymore” (IDI, male, age 18).

Local health professionals also stated that the project lacked psychosocial support and empowerment programs, only covered a small part of the affected population and provided supplies and footwear to relatively few people. The project activities were not institutionalized into local healthcare facilities or other community organizations such as schools. A key informant said, “The intervention helped us to provide some materials for few patients. However, only a limited number of patients received shoes and other support. In addition, we don’t provide counselling services for patients. After the intervention implementers provided the services for three months, the support was discontinued” (KII, local health professional, Yilmana Densa district).

As depicted in Fig 1, the context interacted with the setting and influenced the implementation dimensions of the intervention.

Based on their features, roles and power, each factor interacted with one another and the implemented intervention. We visualize the relationship among each element of the system using a complex system map. Fig 2 illustrates the co-interaction of these factors and the network they formed.

Single arrows, “Forward” indicate the relationship from one component to another.

Double arrows, “Bidirectional” indicate mutual relationship between the components

### The interactive effects of geographical context

Fig 2 shows that geographical context had a direct negative effect on epidemiological factors, lived experience and the outcome of the intervention. Podoconiosis is a result of reaction to chronic contact with red clay soil derived from volcanic deposits. Red clay is the dominant soil in the two communities. Coupled with lack of shoe wearing, this geographical factor heightened the prevalence of podoconiosis, i.e., the epidemiological factor. Scattered settlements affected the way people experienced podoconiosis. We observed that affected individuals travel long distances to farming lands and markets; thereby worsening the way they experienced the disease. Living in remote areas, affected individuals reported that they faced challenges in involving and completing the intervention activities. One example includes, “I can’t travel long distances such as going to the market or visiting relatives in other locations. I also find it difficult to go to healthcare facilities” (IDI, affected female, age 64).

### The interactive effects of epidemiological context

Fig 2 shows that epidemiological context is connected to five different factors, namely, geographical context, socio-cultural context, socioeconomic context, lived experiences, and implementation. Epidemiological context had a bi-directional interaction with socio-cultural context. A key informant said, “The prevalence of podoconiosis and its debilitating physical injury contributed to health-related stigma and widened existing gender inequality in the two communities” (KII, staff of NGO). Affected individuals reported that although unaffected individuals do not tend to discriminate against affected individuals, most of them are not willing to marry patients. Particularly, female patients struggle to find a marriage partner. An affected woman said, “One of the problems I faced was finding a marriage partner. It is not easy to find a marital partner like healthy girls do. I have never received any marriage proposal thus far. No one dared to do so because they don’t want to marry a sick girl like me. My parents often insist me to move around the church, a marketplace, and other public gatherings if I could get one” (IDI, affected female, 35–38 years old). Socio-cultural elements such as beliefs and misconceptions, and lack of shoe wearing practices increased, among other factors, the magnitude of podoconiosis.

The prevalence and impact of the disease affected the livelihoods of affected individuals and their family members. An affected person said, “The disease is painful and sometimes it keeps me at home. During the farming season my sickness leaves me behind from preparing the farmland for the next harvest. Missing out on that period affects my economic life” (IDI, affected male, age 54). Additionally, key informants argue that the poor socioeconomic status of the country, the community and affected individuals heightened individuals’ exposure to the condition and worsened the impact of the disease. One key informant stated, “We live in a very poor country. This is also a rural area, and most public services are not available. Only few individuals have access to basic services” (KII, local official, Yilmana Densa District).

The epidemiological context affected the way affected individuals experienced podoconiosis. The disease forced affected individuals to consider themselves as ‘less able’ to perform social roles. An affected individual said, “Due to my condition, I feel that I am useless” (IDI, affected male, age 39). In turn, individual perceptions arising from their experience with the disease intensified self-isolation and exacerbated the impact of having podoconiosis.

The physical and social impacts of podoconiosis also affected participation in the health intervention. Some affected individuals reported that they could not take part in the intervention due their recurring illness. A quote from one patient reads “The main problem I am often encountering with this disease is the acute attack. It interferes with my daily activities. I can’t travel long distance. I found it difficult to go to the market and healthcare facilities” (IDI, affected female, age 64).

### The interactive effects of socio-culture context

Socio-cultural factors including values and norms, individuals’ status and roles, knowledge, beliefs, attitudes, customs and institutions connected to four domains: epidemiological context, socioeconomic context, lived experience and implementation.

The analysis showed a mutual relationship between socio-cultural and socioeconomic contexts characterized by both positive and negative influences. We observed strong social bonds among community members. Socio-cultural context such as solidarity and community organizations helped residents to cope with hardship during difficult times such as the death of a family member. One affected individual stated, “We have a spiritual association established among close friends and relatives. We use this forum for discussion about our social lives and share information. We have spiritual fathers to give blessings on that day. We also help each other if any members of our association fall in a serious problem. It is also a forum for reconciliation when individuals or families go in conflict” (IDI, a 40-year-old affected male).

On the other hand, some controversial traditional practices such as firing bullets and extravagant expenses negatively impacted the socioeconomic status of individuals as well as the wider community. According to the participants, firing bullets as part of events such as weddings and funerals is a harmful traditional practice that affects people’s lives and financial conditions. An affected woman reported, “Firing bullets is a harmful practice through which we lost many lives in broad daylight. It is also nonsense to expend money on bullets for unnecessary purposes” (IDI, a 38-year-old affected female). In remembrance of deceased members, community members also spend a significant amount of money to prepare a commemorative feast. A participant of FGD said, “When a member of a family dies, the family would feel the pressure of preparing a costly festivity. People should only allocate money that they can afford. Some of the residents borrow money to prepare a feast” (FGD, Women, Dera District). Participants reported that such practices increased household expenditure meaning that residents had little to spend on other needs such as health and education. Poor socioeconomic status could also have a constraining effect on socio-cultural context: due to poverty, some affected individuals reduced their participation in traditional associations.

The influence of socio-cultural factors on the lived experience of affected individuals was both negative and positive. Unaffected community members’ misconceptions such as the belief that podoconiosis is a heritable condition and the resulting negative attitudes towards affected individuals led to low self-esteem among affected individuals. In turn, self-isolated patients had limited their involvement in social activities. An excerpt from one affected person reads, “I feel ashamed of being with people in public gatherings assuming that I become the center of attention due to my abnormal feet. Some friends, priests and kind people invite me in church to sit next to them, but I do feel that my presence next to them may give discomfort to others. So, I don’t usually go out” (IDI, affected female, age 34–36). A key informant said, “Patients show a tendency to self-isolate themselves and avoid contact with others. For instance, podoconiosis patients with infections and wounds never go to churches, as they are required to take off their shoes to enter the church. Patients with infection and wounds may not dare to do so because they are ashamed of the offensive odor that might be disruptive to others. Hence, they would rather stay at home” (KII, NTD officer, Yilmana Densa District).

Some affected individuals reported that social cohesion among community members helped them to embrace their condition and move forward. One affected individual said, “ I have not encountered any problem with regards to my illness from my neighbours. This is just a disease that could have happened to anyone in the community. I think the community members understand this situation very well. I have done nothing to deliberately bring and transmit this disease. Therefore, there is no way that the community could expel or avoid me from social life. I lived peacefully” (IDI, affected female, age 64).

Socio-cultural context also interacted with the implementation of the intervention. Involvement and acceptability of the intervention was affected by socio-cultural contexts such as misconceptions, gender inequality and lack of shoe wearing norms. Beliefs such as that podoconiosis is not a treatable condition prevented some seeking help from healthcare settings and taking part in the intervention. Husbands mostly make treatment seeking-related decisions. Some affected women did not take part in the intervention as husbands did not give them permission. Two examples include:

“I have no one to do the chores when I get sick, so I am supposed to handle the pain and take care of my family. If I go to the health facility whenever I am sick, who will perform the household chores? I didn’t go to the healthcare facility and take the training on self-care management” (IDI, affected female, age 38).

“Husbands remain to be the leader of the family, and all the resources are handled by them. So, wives go to healthcare facilities and buy medicine when husbands allow.” (IDI, affected female, age 64).

### The interactive effects of socioeconomic contexts

Socioeconomic context was related to four factors: epidemiology, socio-cultural, lived experience and implementation. The socioeconomic context had a mutual relationship with epidemiological and socio-cultural contexts. It also had a mutual relationship with lived experience, and a direct effect on the implementation aspect of the intervention.

Socioeconomic context negatively impacted the lived experience of affected individuals. The major livelihood activity, farming, demands physical strength and mobility, influencing the way affected individuals experienced the disease. A few believed that they have become less valuable members of the community as they could not fully engage in farming activities. One example includes, “Had it not been for my pain, I would have become a rich farmer. I can’t work as I wish due to my leg” (IDI, affected male, 39-year). Lived experience also had taken its toll on socioeconomic status. Living with disease for many years, some affected individuals gave up on treatment and opted to stay at home due to low self-esteem, further weakening the socioeconomic status of affected individuals and their family members.

Socioeconomic contexts also interacted with the implementation dimensions of the intervention by hampering the acceptability and sustainability of the project activities. A key informant reported, “As most of the patients are the poorest of the poor, they were unable to afford health resources” (KII, staff of an NGO). Some affected individuals could not continue foot care because they could not purchase treatment materials. One affected person said, “I am poor. I can’t afford to buy shoes and soap if the government doesn’t help me” (IDI, affected male, 39-year-old). Additionally, key informants reported that since the national and regional government could not provide health resources, project activities could not be continued at healthcare facilities.

### The interactive effects of legal context

Legal context is connected to two factors: political context and implementation. The document analysis shows a bidirectional relationship between legal and political contexts. The legal accountability of the Ethiopian government to protect the health of its citizens has enforced ratification of national programs to control podoconiosis. On the other hand, the political commitment to protect citizens from ill health has encouraged ratification of regulations that aim to fulfil citizens’ right to water and sanitation, i.e., the adoption of the Sustainable Development Goals.

The legal context has affected implementation. The staff of intervention implementing NGOs reported that the government’s legal responsibility to provide support for affected individuals resulted in the collaboration of government units at national, regional, and local levels and positively affected the implementation of the intervention.

However, key informants noted that as the government failed to provide treatment supplies at healthcare facilities, it was not possible to provide lymphedema management services or health education programs. A key informant stated, “The intervention was implemented with the assumption that the government offices would allocate a budget for treatment activities. The implementers planned to cover only 20% of the cost associated with treatment and provision of supplies. As this did not happen, the project activities could not be sustained at health care delivery points” (KII, staff of NGO).

### The interactive effects of the political context

Political context is connected to six factors, namely, legal context, context, setting, implementation strategy, implementation process and implementation outcome.

The political context was also in a mutual relationship with the setting of the NGOs. The political administration influenced the organizational culture of the setting. Staff of NaPAN and IOCC stated that they developed activities in line with the country’s proclamation for civil societies. The NGOs actively engage in the development of national programs and guidelines. The document analysis shows that the Ministry of Health, Ethiopia acknowledges the two NGOs for their contribution to the preparation of national programs. The Ministry of Health stated that the second national master plan for NTDs has been prepared in consultative meetings and workshops with several stakeholders including NaPAN and IOCC [5].

The political context had a direct impact on implementation, particularly the implementation strategy. The Ethiopian government developed a national master plan that encourages utilization of local healthcare facilities to provide podoconiosis treatment services [5]. NGO staff reported that this was one of the reasons for shifting from direct, standalone projects to an integrated approach. A key informant said, “The strategy of this project is in congruence with the national NTD master plan that underlines the importance of linking intervention activities with the local health structures” (KII, NTD team leader, Dera District). The political context also directly influenced the implementation process. National, regional and local administrators were involved in launching the intervention and regional health bureau government employees collaborated with the implementers to provide cascade training for health professionals. Furthermore, the political context influenced the outcome of the intervention. The involvement of government offices and local health care facilities was critical in reaching the target communities. A key informant said, “This project wouldn’t have implemented without active involvement of the political elites of relevant government sectors and local health structures.” (KII, NTD focal person).

On the other hand, lack of government budget for lymphedema services exerted a negative influence on service coverage and the sustainability of project activities. A member of staff of the ministry of health reported, “The Ministry has not allocated any budget for podoconiosis in the years 2022 and 2023” (KII, Podoconiosis focal person at Ministry of Health).

### The interactive effects of lived experience

Lived experience interacted with five factors. These included geographical context, epidemiological context, socio-cultural context, socioeconomic context, and implementation outcome (Fig 2). Lived experience was in mutual relationship with implementation outcome. Living with podoconiosis for many years, some affected individuals developed a fatalistic attitude towards the treatability of podoconiosis and showed little interest in adhering to self-care practices. An affected individual said, “It has been many years since I started living with the disease. There is no promising improvement in my condition since I have started the treatments. I gave up hope all together” (IDI, male, age 18).

On the other hand, the intervention helped some affected individuals to accept their condition and reconceive their self-perception - “After receiving the treatment and regularly wearing the shoes, I have seen improvement. Before the treatment, I had a hard time working and travelling long distances. I had recurring pain due to acute attack, and I was not able to work like a healthy individual. Now that the swelling is gradually declining, I can work like anybody else” (IDI, affected male, age 64).

### The interactive effects of the setting of the NGOs

The setting interacted with the political context and had a direct influence on five implementation dimensions of the interventions: agent, theory, strategy, process and outcome. We observed that the setting of the NGOs was mostly characterized by a convivial environment. The offices were quiet and furnished with necessary equipment. The NGOs had clear organizational rules, mission and vision to control podoconiosis. We observed proper documentation of monitoring and evaluation activities. The NGOs used project supervision guidelines to measure the progress of activities, achievements, progress, challenges encountered and feedback.

A handful of staff who had university education and more than a decade of experience were working at the NGOs. We observed the staff interacting in a friendly and respectful manner during office hours. We found the staff to be fully committed and to have a good reputation in implementing interventions. During key informant interviews, we realized that the staff were knowledgeable about podoconiosis, similar NTDs and intervention implementation. The staff perceived their workplaces to be a suitable environment, and the job had become their passion. According to key informants, long years of work in the area made them develop compassion and a heartfelt approach to the suffering of patients. We noted that staff frequently travelled to the intervention sites to meet patients. While staff characteristics and the formal structure of the organizations mostly helped in smooth implementation, the tendency of the NGOs to over rely on checklists limited their ability to independently deliberate on local problems and solutions.

Staff profile and experience, and strategies adopted by the organization influenced the implementation theory, i.e., their assumption how changes could be brought about by utilizing local healthcare facilities and health professionals. Similarly, the organizational culture influenced the project strategy of employing an integrative approach to deliver project activities. NGO staff said, “Our prior experiences in implementing intervention made us realize that integrating intervention activities with local healthcare structures would yield better results than standalone projects” (KII, staff of NGO).

On the other hand, limited funding had a negative influence on actors such as HEWs. The NGOs could not pay incentives to HEWs who recruited patients for the intervention, meaning that HEWs showed little interest in following up the adoption of health messages at household level. Our document analysis showed that the monitoring indicators relied heavily on routine data collected by healthcare facilities. The indicators mainly focused on outreach reports and the NGO staff did not assess whether intervention activities were translated into better understanding of podoconiosis or adoption of healthy behaviour at household level. Relying on a single data source may affect the objectivity of monitoring.

### The interrelationship between implementation domains

The implementation aspect of the intervention has five dimensions, namely, agent, theory, strategy, process, and outcome. On top of being influenced by the setting and the context, these dimensions interacted with one another. The implementation process established connections with five factors: political context, setting, implementation strategy, implementation agents, and implementation outcome. The implementation process was influenced by agents such as donors who mobilized funds for the executed activities, so for example, implementation and monitoring activities were conducted in line with the request of donors. Staff profile also influenced the implementation of the activities. The programs were executed by passionate, experienced staff who worked hard to put the intervention into practice. We saw these staff themselves washing patients’ feet as a demonstration of lymphedema management at healthcare settings.

Implementation agents interacted with five factors. These were the setting, implementation strategy, implementation theory, implementation process and implementation outcomes. Document analysis revealed that agents (donors and implementers) either developed or endorsed a theory of intervention. Having worked for over a decade in podoconiosis prevention and control, the staff of IOCC and NaPAN understood the importance of an integrated approach in bringing about behavioural changes in the target communities.

Implementation outcome was influenced by the setting, political context, socioeconomic context, socio-cultural context, epidemiological context, geographical context, legal context, lived experiences, implementation strategies, implementation agents, implementation theory and implementation process.

### Analysis of major actors in the context, implementation and the setting

We found informal relationships between affected individuals, family members, community members, traditional associations, and religious leaders, and formal relationships between IOCC/NaPAN, district administration and the regional health bureau. For instance, the NGOs signed a memorandum of understanding with the Amhara region health bureau to collaborate on the implementation of the project.

Fig 3 shows that people, civil society organizations, government bodies, healthcare providers and community organizations interacted during the implementation of the intervention. The Health Development Army (HDA) was the exception, and though they were initially identified as potentially important actors by the NGOs, the network was not functional and so did not contribute to implementation of the intervention.

The Ethiopian Ministry of Health plays a leading role providing leadership in podoconiosis and other NTD prevention and control. The Ministry is further tasked with the responsibility of mobilizing resources and assuring the financial sustainability of national NTDs programs. It is also responsible for advocating for NTD programs and enhancing monitoring and evaluation activities. In line with the country’s political administrative structure, NTD programs are governed by the Federal Ministry of Health at national level and regional health bureaus at regional level.

The study found that IOCC/NaPAN collaborated with the Ethiopian Ministry of Health during the planning and implementation of the project activities. The relationship between IOCC/NaPAN and the Ministry was positive. The success of the NGOs in implementing interventions on podoconiosis motivated the Ministry of Health to make podoconiosis a health priority. The government considered the NGOs as major stakeholders in NTD prevention and control (KII, podoconiosis focal person, Ministry of Health). The NGO staff also recognized the positive role of the ministry of health in the implementation of the intervention. Key informants stated that policy makers and staff of IOCC and NaPAN interacted with one another at regular quarterly meetings of the LF and Podoconiosis and WASH-NTD technical groups.

Key informants reported that the NGOs collaborated with the regional health bureau and district level officials during the implementation of the intervention. The staff of IOCC and NaPAN provided capacity building training to health professionals. The regional health bureau interacted with the federal ministry of health: “the regional health bureau attempted to implement national NTD programs by establishing NTD structures and assigning focal person at zonal and district levels” (KII, staff of Ministry of Health). Key informants further revealed that the regional health bureau received technical and financial support from the Ministry of Health. The regional health bureau submitted quarterly activity reports used for decision making by the Ministry of Health.

Healthcare providers and government units also interacted with one another. Key informant interviews revealed that the staff of the regional health bureau provided training and supervised health professionals’ activities. Health professionals and district level administrators met during review meetings and discussed healthcare service challenges at healthcare facility level. Following reports and discussions, the district administration allocated a budget to healthcare facilities. However, key informants reported that health professionals’ requests for funding were not always fully addressed. Some discussions during review meetings resulted in frustration among health professionals as their demands for budget for health resources such as bowls and soap were not met.

Key informant interviews indicated that health professionals at healthcare facilities provided training for HEWs and supervised their activities. Health professionals also facilitated the establishment of patient associations. However, patients reported that they were not engaging in these associations at the time of this study. HEWs conducted health education interventions on communicable diseases, but not podoconiosis at churches and schools. HEWs provided healthcare services to affected individuals, family members, and community leaders at the household level. They also attended social gatherings and traditional association meetings to provide health education. During the implementation of the evaluated intervention, however, their role was mainly limited to recruiting affected individuals.

Affected individuals formed relationships with community organizations and organizations/institutions. For their treatment needs, affected individuals relied on health professionals, HEWs and traditional healers. Religion plays an important role in the lives of rural residents. We observed that affected individuals went to churches to fulfil their religious duty and to seek healing. Affected individuals also relied on religious teachings to embrace their condition and interacted with religious leaders on a near-constant basis. These relationships helped affected individuals cope with some of the challenges related to their physical condition.

Affected individuals reported that they were in constant contact with community leaders and collaborated on community activities like managing wells and mediation ceremonies. Focus group discussions revealed that traditional associations provided economic and social services to affected individuals, family members and community leaders. In turn, these individuals paid membership fees and fulfilled membership obligations, i.e., attending monthly meetings. Traditional associations and churches collaborated to provide burial and other religious services.

After mapping these actors and their relationships, we measured the relationship using degree centrality, closeness centrality and betweenness centrality to identify the ability of each actor to deliver information about health interventions.

The analysis revealed that the set of all people and organizations in the networks reachable via any number of steps was 16. Table 2 shows that patients, health professionals and HEWs have a higher degree of centrality. This means these actors have a higher number of direct ties with other actors in the network indicating they can access or deliver more intervention activities. However, the analysis showed that HEWs were not well connected with the implementers. The HDA, patient associations and schools were the least connected actors in the network.

Closeness centrality indicates the distance each actor has from all other elements. Actors with high scores can reach other actors much more readily. Having higher score of closeness centrality, health professionals (0.594), HEWs (0.552), and community leaders (0.443), occupied a prominent position in the network as they are easily reachable by other actors through a shorter path length.

With, respectively, scores of 0.371 and 0.335 for betweenness centrality, health professionals in healthcare facilities and HEWs are central to their networks. Actors with high centrality are assumed to have a powerful effect as they are considered essential within their networks.

## Discussion

Focusing on a health intervention implemented against podoconiosis in Ethiopia, this study demonstrates the extent to which elements of the implementation, the context and the setting interacted with one another and impacted the intervention. As posited in the CICI framework, multiple contextual factors interacted with one another during the implementation of the intervention. Showing the interactions between these factors is necessary to facilitate an overall assessment of an intervention [9].

Most interactions constrained the acceptability and sustainability of the intervention activities. The rurality and remoteness of the intervention area, the high prevalence of podoconiosis, misconceptions about the disease, gender inequality, lack of shoe wearing practices, low economic status and the Ethiopian government’s failure to fulfil its obligation to protect the rights of patients interacted with one another and negatively affected involvement in the intervention and efforts to sustain the project activities at healthcare settings. On the other hand, political context interacted positively with setting, resulting in expansion of national programs and setting up NTD structures at district level.

Some of the factors appeared to have a more significant influence on health interventions. These factors interacted at multiple levels, i.e., macro, meso and micro and influenced the outcome of the intervention. At macro level, political factors seemed to have a powerful influence on the outcome of health intervention. Additionally, community resources at meso level and micro level agents such as health professionals and patients appear to have affected the implementation and outcome of the intervention to a larger extent.

The political will and supportive government structures have a considerable impact on the continuation of intervention activities [21]. A prior study also showed that contextual factors accounted for inequitable intervention outcomes in four African countries; namely Cameron, Ghana, Liberia and Nigeria [22]. On top of confirming the findings of these earlier studies, our study has highlighted the interactions of political contexts with the setting and implementation factors. This may help implementers to develop a deeper understanding of complex systems. Richer understanding is important to create a healthy system wherein each element plays a significant role in enhancing the prevention and control of podoconiosis. Bringing about changes by utilizing existing structures and actors requires understanding that each actor has their priority, knowledge and power [23]. Considering each element to be interdependent, intervening in one of the factors or actors could change other actors [24]. Improving the responsiveness of these elements to health intervention might therefore result in gradual community change.

The political context influences levels of resources and local health professionals’ range of options to control podoconiosis. Strengthening the formal and vertical relationship between governments and local actors is an important strategy in controlling NTDs. This requires increasing the commitment of each entity to fight NTDs. Political actors may not always be responsive to the demands of local actors. If ‘killer diseases’ are prioritized, governments in LMICs may lack the resources to combat ‘non-killer’ NTDs. NTDs operate in the context of global inequalities. Poor countries should not be left alone to fight diseases like podoconiosis as there is a clear connection between the countries’ deprivation and the wealth of the global north [25]. For instance, Shahvisi and colleagues indicated that the Ethiopian government’s potential to meet local actors’ demand is hampered by the government decision to pay back its high interest debt [25]. Researchers and implementers shall remind the international community that fighting NTDs in LMICs is one way of assuring global equity. NTDs like podoconiosis may exacerbate existing poverty, civil war and migration and so threaten global security. Making governments and global actors accountable to the rights of affected individuals and at-risk populations might result in diversification of budgets and formulation of programs to expand services.

Meso-level factors, i.e., community resources, needs, opportunities and challenges, are important factors that need to be taken into account when designing and delivering interventions. The NGOs failed to tap into some groups of existing actors and networks, for example HEWs, to convey health interventions. HEWs had extensive ties and frequent interactions with local actors, suggesting their powerful potential for information dissemination. This is congruent with findings from Cameroon, Ghana, Liberia and Nigeria which suggested community health workers to be vital actors to link health systems with the populations they serve [22].

The study identified community associations such as the HDA, patient associations and organizations (e.g., churches and schools) as having the potential to extend health promotion activities. However, these were barely connected to the intervention. These structures and actors hold powerful potential to influence health interventions and catalyse positive changes. We made the interaction of community actors explicit to suggest ways of including them in future health interventions. Interventions are likely to succeed if implementers understand the power, interest and network of each key actor and the incentives the actors need to implement intervention activities [26].

HEWs can be employed to mobilize micro level actors such as patients, family members and community leaders. These actors can be more agentic through mutual interaction as they experience emotions which enable them to hear and understand others’ perspectives during interaction [27]. Including health messages in informal interactions creates room for thinking about the suffering of patients and encourages the suggestion of local solutions. Ingraining intervention activities into community structures will also help sustain changes among the actors. If supported, local agents, through their interaction among themselves and with local community organizations, could then institutionalize changes [27].

Designing and implementing the intervention requires appropriate delivery actors [26]. We identified actors who are trusted and respected by community members, who might support control of NTDs. Recruiting local actors into health interventions brings further advantages as they can suggest necessary program adoption and modification due to their leadership qualities [26]. As they are permanent members of the community, interventions co-created and delivered by local actors can be more sustainable [28]. The involvement of community organizations and other preexisting networks in MDA improved compliance because they were able to reach community members effectively and thereby enhance participation [29,30].

Studying the divergent and convergent interests and roles of these factors is essential to identify what can be achieved and what is difficult to achieve via health interventions [31]. Integrating the views of social sciences into intervention evaluation, the study identified factors that can influence health intervention efforts. These factors are mainly manifested in structures, institutions and agents, and we argue that interventions will be more successful if they can facilitate interactions between these factors. Structural factors that operate at macro level such as the political context, and socio-economic, geographical, epidemiological, social, cultural and legal factors are difficult to change within the lifespan of a health invention. Indeed, the impact of structures on the distribution of health resources is widely evident and any expectation to change health inequalities in a short time is unrealistic [32]. However, as noted by Andreas et al [33], institutions hold some potential to influence structural factors in the long run. Working with schools, religious organizations and traditional associations may lead to modification of rules and roles and this to the amendment of individuals’ behavior and potentially even to modification of structural constraints. On top of direct engagement with these meso level factors, empowering local agents may result in changes to community norms and rules in response to their demands.

Employing the CICI framework, the study provides detailed information about how the context, setting and implementation interacted with one another and affected a health intervention and helped identify what worked under which conditions. However, the study has some limitations. We relied on community members’ suggestions of actors without examining the competence and other characteristics of these actors. Further studies are needed to examine the attributes of local actors and identify the best attributes to extend interventions against NTDs. The interaction of the context, the implementation and the setting could be more complex. Our analysis may not exhaustively capture the complex interaction among these elements. Future studies are also needed to measure the influence of each contextual factor and identify the most relevant factors that should be prioritized by policies and implementations that aim to enhance the effectiveness of interventions against NTDs.

## Supporting information

S1 Data Codebook. (DOCX)

S2 Data System mapping. (XLSX)

S3 Data Actors mapping. (XLSX)
