# Supplementary material for: Interaction of contextual, setting and implementation factors on a podoconiosis intervention in Rural Ethiopia: Results from a qualitative study
Source: PLoS One. 2025 Jul 11;20(7):e0328237. doi: 10.1371/journal.pone.0328237 (PMC12250722; doi:10.1371/journal.pone.0328237)
Supplement: S1 Data — (DOCX) [file pone.0328237.s001.docx]

# **Supplementary material**

# The interaction of contextual, setting and implementation factors on a health intervention against podoconiosis in rural Ethiopia

Kibur Engdawork^^[[1]](#footnote-1)^*^, Getnet Tadele^1,^ Gail Davey^^[[2]](#footnote-2)^,3^, Papreen Nahar^2^, Shahaduz Zaman^2^

College of Social Sciences, Addis Ababa University, Ethiopia. ^2^Centre for Global Health Research, Brighton and Sussex Medical School, UK. ^3^School of Public Health, Addis Ababa University, Ethiopia

Thematic text extract selection on context, implementation and setting

| **CICI dimension** | **Construct** | **Theme** | **Key findings** | **Supporting quotes** |
| --- | --- | --- | --- | --- |
| **Context**  Context refers to a set of characteristics that consists of active factors in which an intervention is embedded. (Rychetnik et al. 2002). | **Socio-cultural**  Explicit and implicit behaviour patterns, including their embodiment in symbols and artefacts; the essential core of culture consists of historically derived and selected ideas and values that are shared among members of a group. It not only refers to the conditions in which people are born, grow, live, work and age but also embraces the social roles a human being takes on as a family member, community member or citizen and the relationships inherent to these roles. Constructs such as knowledge, beliefs, conceptions, customs, institutions and any other capabilities and habits acquired by a group are covered by this domain. | **Values and norms.**  **General standards about desirable things in life and specific behavior guidelines in the communities** | **Valued things: Religion**  The majority of the community members are followers of Orthodox Christianity. Religion is highly revered and shapes residents’ beliefs about things. Residents observe fasts and go to church every week to worship. Most people try to explain things including disease in supernatural terms. The community gives high regards to the spiritual structures (associations) and their leaders.  **Desirable norms:**  **The communities have a habit of sharing.**  **Celebrating holidays together**  **Treating patients positively** | - I am a farmer. During the farming season I wake up early in the morning go to church to salute and praise the Lord with prayer and set the oxen for farming and go to my farm field to plough. Sometimes I may go directly to the farm and give prayed at the church I may find on my way to the farm (IDI, affected female, age 64). - We can’t prevent anything if it is the order form God. Anything can happen anytime by the will of God (IDI, affected male, age 63) - People are the creatures of God, and no one is different. Therefor, everyone has been treated as normal human being (IDI, affected female, age 38). - The Lord gives and lord takes away. God gave me a beautiful foot at birth, and HE took it away. I have not taken remorse against God. I thank my God because he also gave me the strength to cope up the challenges (IDI, affected female, age 35 to 38 years old). - I think the best solution is to teach the community through religious institutions and structurers. ..people have more respect to their religion and to the religious leaders (KII, HEW, Dera District). - In fact, we have no unique culture that is different from other woredas. Sharing is a norm in our culture, it is a longstanding norm passed on from generation to generation. We eat and drink together helping each other (FGD, Women Yilmana Densa District) - Hospitality is another good culture in which we treat strangers. In the rural area, it is a common practice to welcome strangers at home with food and coffee to the extent that strangers feel at home. This is a promising culture which should be promoted (FGD, Women, Dera district) - I know that there are some volunteers and rich individual who are helping patients during holidays. They provide them with financial and material assistances to the poorest of the poor patients in our community, so that they can enjoy the holiday with their family. Not all patients are benefiting from this but only those who are the poorest and those who cannot afford for holiday expense (FGD, Women Yilman Densa District) - We have a common religion i.e orthodox and we have a culture of inviting over people for food and drink specially during holidays. This is particularly common after the easter holiday. We enjoy eating and drinking together with relatives and friends. We also have spiritual association established among close friends and relatives. We use this forum for discussion about our social lives and share information. We have spiritual fathers to give blessings on that day. We also help each other if any members of our association fall in a serious problem. It is also a forum for reconciliation when individuals or families go in conflict (IDI, a 40 years old affected male) - The community members are more considerate to people affected by podoconiosis. Patients have never received any bad treatments form the community members. They live together in harmony and participate in any social life just like any other healthy individual. There is no discrimination against patients. That is one of the good things I observed in our community. In fact, some people might be reserved to come closer to patients in the interest to avoid bad smell of the feet. Other than that, they face no challenge in this regard. They are not stigmatized at all. I have not heard of any case being stigmatized or harshly treated in the community. However, the main challenge most patients face is mobility. It highly affects them form performing their daily routines and social activities unlike the healthy people. They cannot take a long-distance walk. They also have a serious problem of getting shoes that is suitable for their feet as the swelling doesn’t allow them to wear a regular size shoe (KII, HEW, Yilmana Densa District). - I have lived my age in this community and have never seen any bad treatment against patients affected by disease of any kind. I have not encountered any problem with regards to my illness from my neighborhoods in my village. This is just a disease that could have happened to anyone in the community. I think the community members understand this situation very well. I have done nothing to deliberately bring and transmit this disease. Therefore, there is no way that the community could expel or avoid me form social life. I lived peacefully and with good social integration (IDI, Affected Female, age 64) - As far as I know, no one stigmatizes anybody. All people are treated in the same way. It is not in the nature of our community. I haven’t encountered any of such people. People are the creatures of God, and no one is different. Therefore, everyone has been treated as normal human being. I have never encountered any. We are surrounded by close relatives in my villages. They are considerate towards me. In fact, when I encountered in conflict with a non-relative, they may use bad words to insults me referring to my illness. Other than that, there is nothing serious happened to me so far (IDI, Affected female, age 49) |
|  |  |  | **Shoe wearing practices.** The participants argued that shoe wearing practices is becoming a norm among the younger generation, but still most old people walk barefoot. | - Young people never go bare feet. Only the people older than me who would like to be bare feet. Particularly walking bare feet is commonly observed among women. I mean in terms of proportion it is the highest among women, but men also do the same specially on farms (FGD, Men, Dera District). - In the past, there was no shoes at all. It was on considered as an important item. Only a few people were using footwear. After the health extension program came into a picture in late 1990’s, people started to wear shoes. people started to consider shoes as important item. It is improving but still the practice is inconsistent. Specially people in the rural community do not wear shoes at home or around villages and in farm activities. Most people wear shoes when they travel long distance. In fact, young people are better in this regard, but not conclusive. Still many young people also spend bare feet at home and in the farm fields (KII, regional health bureau officer, Dera District) - I think there is a lot of change in shoes wearing practice among the youths and adults. Old people stick to their old tradition and walk bare feet. Improvements in the economic capacity of families has also contributed to the change in shoes wearing practice (FGD, Women Dera District). - Today there is improvement with regards to the shoes wearing practices among young people. Except during the rainy season (where almost all people opt to walk bare feet when it is muddy, assuming not comfortable to work) young people wear shoes all day and every day (KII, HEW, Dera District) |
|  |  | **Relevant changes in norms** | **From homebirth to health facility delivery.** Respondents believe that women in the community are delivering at healthcare facilities following the health education and follow-ups by HEWs**.**  **Seeking treatment from health care facilities.** The respondents believed that residents are increasingly visiting healthcare centers to seek treatments.  **From open defecation to latrine**  Community members reported that defecating in fields and forests had been a common practice as there was a lack of toilets.  **Improvement in women status.**  Community members reported that women’s right have been improved recently following legal interventions. | - A number of women used to give birth at home and some died due to heavy blooding during labour. We can say this has been changed now as women give birth at health stations (FGD, Women Yilmana Densa) - Thanks to health professionals we are not using latrines. Back in days we used to defecate wherever we want. Now we even ridicule people who defecate in the fields (FGD, Men Yilman Densa)   Unlike the previous times, people now have a community-based health insurance and never hesitate to come to the health center whenever they feel pain. Earlier, people hardly visit the health center except for sever cases. Now they visit the health center for any type of disease and could be referred to higher level medication at woreda or in big towns like Bahirdar( FGD, Women, Yilmana Densa District).  Nowadays, women have the right to acquire and use lands and properties. If they get divorced, they will take equal share of the lands and properties. Women have right to hold the right of child custody after divorce. Back in days ex-husbands didn’t support ex-wives. Nowadays every divorced husband financially supports their ex-wives. This is due to law(FGD, Women Dera District) |
|  |  | **Harmful and disliked activities** | **Stray bullet.**  A number of individuals have complained about men’s habit to fire bullets during funeral and wedding ceremonies. Respondents reported that the habit resulted in unintentional and accidentally killing of residents.  **Blood feud**  Respondents discussed that avenging the blood of a murdered family members is a common practice that resulted in endless feuds among families and relatives.  **Extravagant expenses.**  In fond remembrance of deceased members, the community spends a significant amount of money to prepare a commemorative feast.  **Undesirable norms**  **Insulting patients.**  It was reported that in some instances patients are subjected to insults by unaffected members of the community.  **Early marriage**. Some respondents stated that some girls are still forced to marry at early age.  **Evil Eye.** The community believes that evil eyes, people who possess special power causes illness and even death are the most disliked and marginalized people. The community folk informs that evil eye (buda) are capable of transforming themselves into hyenas at night. | - The most harmful practices is firing bullets during weddings and mourning ceremonies. People fire bullets in these ceremonies to express their condolences over the death of someone or happiness on festivities. It is a dangerous practice because we lost many lives with reckless firing. Some people fire billets to test their guns and the bullets may go to a different direction and kill people. There are incidents where the bride and groom were killed on their wedding by such wild firing (FGD Men Dera District). - Gun fire on morning ceremony is also another thing that I dislike most. This is a harmful practice on which we lost may live in a broad day light with reckless shootings. It is also nonsense to expend money on bullets for unnecessary purpose. These days farmers in our community give more value to guns than any other property. In our community, a person is assumed to be rich if he owns a gun than plots of land or grinding mills (IDI, a 38 years old affected female)   Another long-standing cultures that should be avoided is the blood revenge practice. Many people have lost their lives due to this bad practice. As far as I am concerned, the most harmful practice is blood revenge killings. It creates disaster in families. Some families will evacuate form their birthplace due to fear of retaliation. While most harmful practices such as child marriage are declining, the practice of blood revenge has not been changed in our district. The practice is continuing. No action has been taken so far to avoid this bad practice (FGD, Men, Dera District)  When a member of a family dies, the family would feel the pressure of preparing a costly festivity People should only allocate money that they can afford. Some of the residents borrow money to prepare feast (FGD, Women, Dera District)  • Patients attend all social events without discrimination, but people may insult them or humiliate them and or show aversive reaction towards their feet (FGD, Women, Dera District).  • People have unhealthy attitudes towards my feet. Sometimes they call me disfigured when we are sitting together in our public meetings or social gatherings, I see some people looking at my swollen feet in a strange way showing disgusted expressions. Others also make fun of me. Sometimes I feel down and angry. Even my healthy brothers, my elder brothers also mock at me and insult me. One day I gave crushing blow to my elder brother when he insulted me as disfigured and I am not in good terms with him and his family for many years (IDI 19, a 38 years old affected female).  • Some people call us disfigured…. This could have happened to anyone. People should avoid insulting patients. I sometimes hear some people insulting patients. Patients cry and feel down when insulted. [how about you?] The same holds to me. It burns me inside. Disrespecting patients is hurting. Such bad attitude pushes them to commit a revenge (IDI, Female, 64 years old).  I dislike some of our traditional practices such as early marriage specially girls because they are exposed to fistula. The other practice which should be condemned is female genital cutting which his common in our community, which is a dangerous practice persisted for long (IDI, an affected women age 38).   - Individuals with evil eyes are the most stigmatized. People in our community distance themselves from such individual because evil eyes are assumed to have special power to make people sick or die. Our society does not make any social relationship with such people. If they are identified, they will be avoided form spiritual associations and form idir (traditional association) as well. There is no medicine for evil eye. People are afraid of contact with such people for fear of the risk of attack (IDI, women Yilmana Densa). - I think the most discriminated are people with evil eyes People with evil eyes can hurt humans with a devil power in their eyes. They hate sick people. Evil eye itself is an incurable disease and that is why people avoid them from social support in “idir” and” mahiber” (membership in religious associations) and other social events. Specially, their bad spirit kills nice looking people (IDI, Affected female, age 39) |
|  |  | **Social control. The community mainly informal ways to enforce its norm**s. This includes sanctions, which may include shame and ridicule. | **Reintegrative shaming**  The community shows disapprovals for individuals who have committed crime to make them remorse their misconduct that would be followed by reacceptance.  **Exclusion from social activities**. Individuals who violated social norms such as committing robbery will be excluded from social activities. | Our community isolates individuals who often get in conflict with other people and those who are involved in theft /robbery. Such people are excluded from social participations and the community support schemes such as during group farming, harvesting, etc., However, if these individuals correct their misconducts and publicly ask for apology, they will rejoin the social participation (FGD, Men Yilmana Densa District).  Thieves or robbers are also cursed and avoided by the community. They have no membership in idir or spiritual associations. But they live in the community like any other individual (FGD, Women, Yilmana Densa District) |
|  |  | **Social capital.** The participants are members of traditional/social institutions such as *idir* and *mahiber.* They believe that their fellow members will extend their help during their difficult times such as a death of a family member. | **Idir** . the community rely on a self-help voluntary association that serves as economic and social insurance at times of death and other crises. As it stands, idirs only provide help to members during crisis. They don’t serve as platform to help members during sickness or other needs (e.g. clothing) .  **Mahiber:** Spiritual gatherings under the informal association named mahiber are also common practice in the communities. | - Everyone in our community is a member in idir. It is established by the community to support and assist people in need specially helping the poor. It is outside the government structure. It involves all the community members (FGD, Men Dera District) - Idir is a crucial social institution which is useful to strengthen our social bond. It serves as a social insurance for the poor. In time of death(mourning), idir assists the deceased family equally regardless of their wealth status (FGD, Women Yilmana Densa) - People are also organized in idir to help each other in time of mourning when someone passes away. The purpose of idir is only to facilitate the funeral ceremony not helping people in other aspects of life. idir provided some money for the family who lost life. - We commemorate saints on monthly basis under our mahibers (religious associations). This is for our soul and body. By doing so, we perform our spiritual role. In addition, we will have intimate relationship. We talked about our personal problems when we meet (FGD ,Women Dera District). - I am a member of the association that honors saint Gabriel. I am the only patient in our association. The association has around 40 members. I usually participate, except when I am hit by acute attacks. I stay at home when the acute attack arises. It is helpful specially in time of harvest and farming or building a house. When I request the support from members, they are willing to assist me in labor (IDI, a 57 years old affected male) |
|  |  | **Self Isolation** | Some patients prefer to stay at home due to fear of public reaction. | - I feel ashamed of being with people in public gathering assuming that I become the center of attention due to my abnormal feet. Some fiends, priests and kind people invite me in church to sit next to them, but I do feel that my presence next to them may give discomfort to others. So, I don’t usually go out (IDI, affected female, age 34 to 36). - In fact, I sometimes feel that this condition might create discomfort among people. I would like to stay at home specially when the swelling creates wounds and bad odor. (IDI, Affected female, age 65) - Patients show a tendency to self-isolate themselves and avoid contact with others. For instance, podo patients with infections and wound never go to churches, because normal procedure, they are required to take off the shoes to get into the church. Patients with infection and wounds may not dare to do so because they are ashamed of the offensive odor that might be disruptive to others. Hence, they would rather stay at home (KII, NTD officer, Yilmana Densa District) |
|  |  | **Preventive behavior** | Although improving overtime, shoes wearing practices in these communities are highly intermittent. Farming barefoot continued as longstanding norm among adults due to social pressure and wrong beliefs about farming with shoes in general and consistent shoes wearing in particular. | - Farmers are working in their farm bare feet, and it is humiliating to work with a footwear. This tradition is still there (FGD Men, Dera Disrict). - No. I have never worn shoes. In my childhood time, we used to walk bare feet everywhere. Shoes wearing is a recent practice. I started wearing shoes after being affected. Now I have shoes, but I often wear open plastic shoes. Closed shoes are not comfortable for work. I use closed shoe when I travel on occasions (IDI, Affected female age 34 to 36). - Nowadays everyone in the community wears shoes. In my view, about 80-85% of the people in our community wear shoes. In fact, this is a recent phenomenon due to the advances in technology, education and people are now getting more profit form their production and have better savings to fulfill their needs…Except a few people, most of the farmers do not wear shoes while working in the farms because they find it hard and uncomfortable. Other than that, everybody wears shoes when walking everywhere (KII, local official Yilmana Densa District) |
|  |  | **Knowledge about podoconiosis** | Skepticism about treatability of podoconiosis is rampant. Beliefs on traditional and spiritual treatment predominantly present in the community influenced health seeking and adherence of patients to the modern treatment procedures. | It is not worthy to send money for medical treatment on such disease which cannot be cured without the interference of God. I should wait for God’s response to my prayer” (IDI, Affected female, 38) |
|  |  | **Health related stigma**. Some people could face stigma due to their health conditions. Stigma towards patients with podoconiosis is present, but not considered as pressing issue in the community compared to other stigmatizing diseases or problems ranked top in the list, such as Evil eye, fistula, and TB. | HIV/AIDs and TB. People who are affected by HIV/AIDS and TB are stigmatized by the community due to fear of contracting the diseases.  Stigma towards podo patients is present, but it doesn’t entail discrimination or social isolation of patients based on their disease status except in circumstances related to mate selection and marital issues for fear of hereditary transmission. Unaffected community members do not consider this as a problem. Economically better of patients and male patients have a better chance to get married. | - I would say HIV/AIDS is the most stigmatizing. It is a scary disease as it does not have a cure. It is not like cholera that affect everyone. It affects only those who do not take care of themselves. HIV/AIDS kills once it affects the person. Hence people are afraid of creating contact with people with HIV (FGD, Women Yilmana Densa District) - HIV/AIDS is highly stigmatizing. People distance themselves from persons identified or suspected as AIDS patients. The same holds true for TB patients because it is highly communicable. People recovered from TB are also target for stigma despite they are healthy and cannot transmit the disease. Once someone is labeled as TB patient, they are stigmatized despite they are cured (FGD Men, Dera District). - After being affected, I proposed marriage but was unsuccessful for 13 times. I tried my best to get married but all the girls whom I proposed turned down the proposal. Some of them started to live with me for short time and left me as soon as they realized I am affected by this disease. Usually, I cover my feet with long trousers and boots, and they did not notice that I am patient. They usually find out while I take off my trouser at night and they run away the next morning (IDI, affected male, age 60). - One of the problems I faced was finding a marriage partner. It is not easy to find a marital friend unlike healthy girls do. I have never received any marriage proposal thus far. No one dared to do so because they don’t want to marry a sick girl like me. My parents often insist me to move around the church, a marketplace, and other public gatherings if I could get one. But I have already gave up doing this and casted this problem on my God hoping that HE will answer to my prayer related to the marital question one day. There are patients who got married and I hope that God will make it happen to me too (IDI, an affected female, age, 35 to 38 years old). - I know people who have been left behind marriage because of their condition [podoconiosis]. Specially women. Men have a chance to get one. Women are less likely to be chosen by male partners if they are podo patients. I know girls who have not yet married although they are in a reproductive age. I have never raised or discussed such issues with them. I don’t want to do so because it may affect their feeling. Nothing has been done by the community, the government or non-government body to tackle this challenge. - There are many patients in our community. Patients find it hard to marry healthy individuals. But they can marry each other. Why would it bothers you? (FGD, Men, Yilmana Densa District). - Healthy families do not allow their family member to establish marital relation with podo patients. They consider them as physically disabled. Mostly such people get married to each other. Especially sick women have no chance to marry a healthy man. However, men can marry any woman regardless of their physical status. At least they will get a poor woman as last choice (KII, HEWs, Yilmana Densa District). - My marriage proposal was accepted because her family had confidence in my economic capacity. (IDI, affected male, age 50) |
|  |  | **Gender inequality** | Women experienced the greater share of podoconiosis burden due to the existing socio-economic inequalities and gender inequality in the community. This was particularly manifested in their illness experience delayed care seeking and marriageability issues. | - I sometimes face acute for a couple of days. I try to sleep it off. But I can’t rest for long. I have no one to do the chores when I get sick, so I am supposed to handle the pain and take care of my family. If I go the health station whenever I am sick, who would perform the household chores? (IDI, Affected female, age 38) - My husband is also a patient. He went to a healthcare station and received soaps and shoes. The HEWs told him about this but he went there without telling me. My husband took antibiotic when he has acute attack. But he doesn’t give me medicine when I get sick (IDI, Affected female, age 39). - …if both (husband and wife) are alive, the husband remains to be the leader of the family and all the resources are handled by him. So, females go to healthcare stations and buy medicine when the husband allows (IDI, Affected female, age 64). - The other problem I faced was finding a marriage partner. It is not easy to find a marital friend unlike healthy girls do.” (IDI, affected female, age 35) - The prevalence of podoconiosis and its debilitating physical injury contributed to health-related stigma and widened existing gender inequality in the two communities” (KII, staff of NGO). |
|  | **Lived experience**  Lived experience includes the representation of the experience and choices of affected individuals and the knowledge/perception they gained from their experience. This has not been explicitly included in the CICI framework. Understanding the experiences and the world of affected individuals is important for implementors to improve experiences at the point of care and provide directions for quality improvement of intervention programs. | **Daily experience of the patient** | Most of the participants rely on farming which usually requires a physical presence of people. Ploughing, managing cattle and performing other daily activities could be physically charging and affected individuals’ daily activities can be affected by their chronic condition. To some extent, patients want to be cured so that they could participate in their daily activities. | - I will play an elderly role in the community, go to market to sell farm products, attend the mourning ceremony if any or stay at home if I have nothing to do. Now, I am getting weak due to age and illness on my feet. I am not as active as I was before (IDI, affected male, age 60) - Earlier I used to tend cattle. I lead cattle to a field and stay there herding them through the day. My feet were not as severely affected as it was like this, but it was painful when I walk far a distance. Now that, our cattle have been sold out. What is a farmer without it’s cattle? We have only an oxen rented out to other family. Therefore, mostly I stay at home (IDI, affected male, age 18). |
|  |  | **How does affected individuals’ world present itself.** | Podoconiosis transforms the meaning of body for patients. Affected individuals perceive their body in line with inability to function in life. And their goal is to get back to normality. They tend to measure progress as opposed to get normal. If not, their condition reminds them what they have lost. | - When it started, it was itching my feet and toes so seriously. Then gradually wounds appeared on my toes and around my feet. The blood and pus discharging from the wound made it worse and painful. The swelling gradually grew larger, leaving me to the extent that I couldn’t be able to wear shoes to cover it. The swelling is not too large but wounds around my toes gave me a hard time to wear shoes. (A 38 years old affected female). - My feet were so beautiful before the disease had started. I used to walk to fetch water from the river as there was no communal water pipe nearby our village. It was fine but suddenly, an unbearable itching feeling began around my toes, and it made me scratch them frequently. Sometimes I used to scratch with rocks and on any other rough objects. I sought medical treatment, but it was in vain. As time goes by, my feet swollen up and gradually grew bigger and bigger and infected with worms. The fluid squeezing out of my feet was so stinky. To avoid the intolerable itching feeling and offensive odor, I used to spray perfumes and insecticides to refresh the bad smell. Although the swelling is still there, I can’t walk long distance and wear fully covered shoe as it does not feet to my feet. I feel pain when I travel long distance (IDI, affected female 34-36). - Health staff told me that it is curable, but I don’t believe it is true. There is no promising improvement in my condition since I have started the treatments. I gave up hope all together. (IDI-12, male, age 18)/ - Although I tried to wash my feet and bandage it for a few weeks, it was useless. I have not seen any change on the condition of my feet (IDI, affected female age 50). - I want to ask you if I could get support to get a cure from this disease (IDI, affected female age 64). - During the farming season my leg leaves me behind from preparing the farmland for the next harvest (Affected Male, age 50). - Had it not been for my pain, I would have become a rich farmer. I can’t work as I wish due to my leg. (a 39-year-old affected male). |
|  |  | **How does affected individuals reconceive their values in light of their condition** | Respondents also shared their stories that show acceptance of illness. Some of them are no longer trying to get cured and accepted their condition. They are learning to manage acute attacks. | - I used to take tetracycline to get relief form the acute attack. I am fine with taking this medicine. It is only the acute attack that prevents my routine activities. I still cook and clean. I am happy whenever there is food and drink at home. (IDI, affected female, age 64). - They [health professional also informed us to wash feet regularly and apply the ointment and the bandage regularly and properly on our feet. They also advised us to elevate our feet when lying or sitting at home. After receiving the treatment and regularly wearing the shoes, I have seen improvement. Before the treatment, I had a hard time to work and travel long distance. There was a recurrent pain due to acute attack and I was not able to use my potential to work like healthy individuals do. Now that the swelling is gradually declining, and I can work like any body else. My feet have no wound or bad odor as it was before, although the swelling has not completely cured (IDI, affected male, age 64). |
|  | **Epidemiological context**  The epidemiological domain refers to the distribution of podoconiosis in the study area and the attributable burden of the disease as well as determinants of needs of people directly and indirectly affected by podoconiosis. | **Podoconiosis prevalence** | Podoconiosis prevalence rate in Amhara regional state and intervention sites is high. | Macro: In Ethiopia, 345 districts were found to be podoconiosis endemic areas. Among the 164 districts of the Amhara region, 64 are podoconiosis-endemic and 4% of the population is affected (Deribe et al. 2015).  Meso:   - West Gojjam and South Gondar Zones, areas where the selected intervention is being implemented, are among the most highly affected areas as 5-10% of the population is affected by podoconiosis (NaPAN & IOCC, 2020). - Before the intervention, this was a huge problem in the district (KII, NTD officer, Dera District) - We don’t have clear evidence or data on the magnitude of podoconiosis in our district. We have not done any assessment or baseline study, or research done by any other organization either. However, I heard in our training that the prevalence of podoconiosis in our woreda is estimated within 7-10% range (NTD officer, Yilmana Densa District). |
|  |  | **Social and economic impact** | Podoconiosis imposes a huge social and economic challenges on affected individuals and family members. | - **Marriage:** - Since I am a patient, I have never thought about proposing marriage yet. For me, it is hard to find female partners as families are not willing to accept marriage proposal from a person like me. No matter how partners love each other the girl’s family are reserved from giving their daughter to a patient family. This is a big challenge I may face when proposing marriage. I always regret on this matter for lagging behind my agemates and friends (Male affected, age 18)   **Education:**   - Yes, I attended grade one, but soon quitted school because of my sickness. I couldn’t bear the reaction of school children as everybody was pointing their fingers at me with insults (Male affected, 18 years old) - I am left behind all my friends and agemates in education. I have never gone to school for fear of stigma and avoid insults from friends. This is the biggest missed opportunity in my life. all my siblings have completed school and some of them are leading independent life (Affected female, age between 34 and 38)   **Livelihood**:   - During the farming season it leaves me behind from preparing the farmland for the next harvest. Missing out that period affects my economic life (Affected Male, age 54). - **Participation in community activities:** - I don’t participate in funeral ceremonies as I want due to my illness. I have also stopped going to churches. - I had been late to court appointment and funerals due to my illness. I have arrived at cemeteries after burials ceremonies are concluded. I felt very ashamed as I couldn’t pay my respect to the deceased. This is an embarrassing disease.   Affected male, age 58) |
|  |  | **Physical impact** | Podoconiosis affects the mobility of patients. Patients also face frequent acute attacks characterized by chills and fever. | - I was frequently suffering from an acute attack. When acute attack arises, I feel cold inside and pain around the groin. I also sleep the whole day or a couple of days and weeks until the acute attack subsides. That is the worst part of this illness (Affected Male, age 64). - It has been four years since I started having frequent acute attack. Every time I face acute attack, I have waist and muscle ache; whole body becomes weak. I develop chills and I suffer like an old person. The acute attack gets worse during the cold season (Affected male, age 40). - She [a patient] could not take care her child properly due to frequent pain [episode of acute attacks]. Sometimes she can’t breastfeed her baby for two consecutive days (a mother of an affected female patient, age 65) - The main problem I am often encountering with this disease is the acute attack. It interferes with my daily activities. I can’t do my domestic work. I can’t fulfill my responsibility at home. I can’t travel long distance (such as going to the market or visiting relatives in other locations) (Affected Female, age 65)/ - Compared to healthy people, we are useless. My leg doesn’t take me forwards. It holds me back. When we travel as a group to markets or other places, I stay behind. When people reach to their destination, I could only reach the middle (Affected male, age 39) |
|  | **Geographical context**  The geographical characteristics refer to the broader physical environment, landscapes and resources, both natural and transformed by humans, available at a given location. As such it also comprises the infrastructure at a given location, which could result in geographical isolation. | **Rurality** | Intervention was conducted in 7 selected districts located in West Gojam (Yilmana Densa, Jabi Tihenan, Konji Kolelea, Bure Sekela districts), and South Gondar Zones (Dera Fogera districts) in the Amhara region. All the intervention sites are in the rural settings which were identified based on the criteria of high podoconiosis disease perveance | We have been implementing this project for the last three years in selected 7 rural districts in the Amhara region (KII, NTD focal person, Dera District) |
|  |  | **Soil type** | The two districts’ surface areas are mainly covered by a red clay soil which is responsible for the development of podoconiosis. | - The other characteristics that it shares with neighbouring areas is the nature of the soil. In our neighbourhood, most of the land is covered with red soil. If we look at it from the productivity angle, some crops grow well on red clay soil than in black soil. The red soil is good to produce corn and beans, but not good for teff and grass pea. That is the advantage and disadvantage. I see no disadvantage from the health angle except the dust from red soil changes the skin colour of our feet. There is no other disadvantage I would mention (Key informant, local official Yilmana Densa district) |
|  |  | **Remote area** | The residents in the two districts live in remote areas and travel long distances to farmlands, markets and health stations on foot. Some walk barefoot; that could increase their susceptibility to develop podoconiosis. Moreover, their distance from healthcare stations discouraged them from seeking treatment and talking part in interventions. | - I travel a lot for my farmlands are located far from my residence because the area in my residence is rocky and infertile. Therefore, I must travel long distance carrying the plough, crossing up hills and mountains every time to work on my farm (Affected male, 60 years old). - Patients need to travel from remote areas to our health stations. We don’t admit patients for treatment, and they are forced to stay with their relatives who live nearby or go back to their houses after treatment (KII, podo focal person, Dera District) - As I walk a lot long distances on foot, anything that hits my feet triggers pain and acute attack (Affected female, age 38) - I went to the health station as we were told by the HEWs a new medicine has arrived. The health professionals showed us how to take care of our feet and provide us with soaps. I didn’t go in the second round. Why would I travel with my ill leg for three bars of soap? (A 45 year old affected man) |
|  | **Socio-economic context**  The social and economic resources of a community and the access of a population to these resources. | **Poor country**  **(Macro level)** | Ethiopia is one of the least developed countries. Its citizens are prone to podoconiosis due to poverty. The countries low social economic status means, the government has a limited capacity to provide healthcare services for patients. | - Considering health, education and standing of living, UNDP (2022) estimated that about 69% of the population in Ethiopia is multidimensionally poor while 18.4 % were found to be vulnerable to multidimensional poverty in 2020 (UNDP, 2022). Poverty is much more prevalence in rural areas than urban areas in Ethiopia (Forum for Social Studies, 2009). - High level of poverty, civil war and dire economic situation meant, Ethiopia has had to rely heavily on foreign aid. In 2020, the total amount of development aid Ethiopia has received amount to $5,.3 billion making the country among the top five recipients of foreign aid around the world (World Bank, 2022). Foreign aid has been the major government revenue and reported to have positive role in the country’s infrastructure development and economic growth (Girma and Tilahun, 2022; Setargie, 2015). |
|  |  | **Community resources (Meso level)** | Public resources such as running water, electric power and paved roads are rarely available in the communities. Most of the residents rely on communal hand dug wells for drinking water supply. The people also fetch water from rivers.  Health station and primary level educations are found in the selected areas. Most of the residents are enrolled to a community-based health insurance scheme.  Communal natural hot tubs . Some members of the community use natural hot tubs to treat their health conditions. Podoconiosis patients also visit these places to soak their feet.  Traditiaonl healers.  Some patients report to visit traditional healers. We observed that traditional healers provide their servies. But people don’t feel comporatable to mention ther visit to traditiaonl healers | - This is a rural areas and most public services are not available. A few individuals and small-scale millers have access to electricity. Most of our residents have access to communal wells (KII, Local officials, Yilmana Densa District) - I like the community-based health insurance introduced recently for farmers. It gave me the opportunity to receive medical treatment anytime we get sick. It made all the farmers to be treated equally whether you are rich or poor (Affected female, age 38).   I will send them to the hot spring water after applying the herbal treatment, but not before the treatment. The hot spring helps them to release the vessels relax their muscles and to make the wound dry and recover quickly (KII, traditional healer).  People also |
|  |  | **Major livelihood activities (Meso level)** | A great majority of the residents in the community survives on farming activities. | - Our district is mainly known for its agricultural production The major cultivated crops are beans, teff, barely, wheat and maize. Most of the (KII, local official) |
|  |  | **Living in poverty** | Affected individuals come from households with low socio-economic status The main economic activity of the intervention communities is based on traditional farming. However, podoconiosis posed a reciprocal influence on the economic status of patients which in turn affect patients to consistently take up the self-treatment. | - As most of the podo patients are the poorest of the poor, they were unable to afford medical expenses (KII, staff of an NGO). - I am poor. I can’t afford to buy shoes and soap if government doesn’t help me. It is my pain that made me poor. It is only my pain that held me back. Had it not been for my pain, I would have become a rich farmer. I can’t work as I wish due to my leg. (a 39-year-old affected male). - Whenever I have the acute attack, I sleep it off. I can’t afford to go to a health station. I can’t afford the transportation cost. My husband takes antibiotic, but I don’t. I don’t have money to buy antibiotics from pharmacy (An affected female, 32 years old). - “I am a farmer… The disease is painful and sometimes it keeps me stay at home. During the farming season it leaves me behind from preparing the farmland for the next harvest. Missing out that period affects my economic life.” (IDI, male, Agita Keble) |
|  |  | **Poverty and stigma** | The economic challenges intensified self- isolation of patients form participation in social events. Compounded with the increasing cost of living, some patients withdrew from hosting spiritual gatherings due to lack of resources. | Especially those with low economic status are at high risk for stigma. Therefore, the project should have done more on enhancing the economic capacity of patients to minimize stigma. (KII, IOCC staff) |
|  |  | **Living condition (Micro level)** | On a typical day, the study participants spend most of their time performing household chores, and agricultural and related activities. | Usually, I get up early in the morning and clear home and fetch water from the local hand dug well. After fetching water, I prepare breakfast for the family. Then I spend the rest of the day doing all the family chores that women are supposed to do. Cooking, serving food to family, washing, cleaning rooms, and helping husbands in the farms during weeding and harvesting times. Every Wednesday and Saturday I will go to the market at Adet market to buy some items for the family. It is also not the responsibility of women to buy and sell cattle. My husband will do that. I cannot farm in the field. Ploughing is also for men not for women. It is not common to see women tilling land with oxen. It might be hard for women to push and pull the plough (Affected female, age 45)    I am a farmer. All my life issues circulate around farming activities. During farming season, I spend most of my time in the farm field ploughing, seeding, weeding, and harvesting. On other seasons, I look after my cows and oxen taking them to the grazing land. These are my typical duties. On Saturdays and Sundays, I do not work on farming related activities. Saturdays and Sundays are considered as holidays according to the culture of our community and everyone is off work. We go to marketplaces to churches and visiting relatives on weekends. There are also religious holidays every month during which we honor saints. On those days I never work, never cut woods, and engage in any economic activity. I also participate in any social activities such as farmers meetings, spiritual associations, wedding and mourning ceremonies in our neighborhoods or in locations far away from our village depending on my family relationship (A 50 years old affected male). |
|  | **Political context**  The political domain focuses on the distribution of power, assets and interests within a population, as well as the range of organisations involved, their interests and the formal and informal rules that govern interactions between them. | Political structure (Macro level) |  | Ethiopian constitution adopted parliamentary system of government in the country. The parliament has two chambers: the house of people’s representatives and the house of the federation. The constitution further introduced a system of Federalism as a system of government in the country (FDRE, 1995). Ethiopia is composed of 11 ethnically defined states and the two city administrations: Addis Ababa and Dire Dawa that are organized into zones, districts and kebeles (the smallest administered units).  The Ethiopian Ministry of Health plays a leading role in mental health leadership and governances. The ministry is responsible for ensuring the sustainable supply of medicine, securing financial resources and strengthening the research, information, monitoring and evaluation systems for mental health. In addition, the ministry develops and revises national plans and identity target of interventions (FMoH, 2016). In line with the country’s political administrative structure, the mental service delivery system is governed by the Federal Ministry of Health at national level and regional health bureaus at regional level. The mental health program is placed under the Disease Prevention and Control Directorate in the MoH with focal persons for mental health in the district health offices (FMoH, 2016). |
|  |  | Political commitment to fight podoconiosis | Ethiopian government has shown commitment to fight podoconiosis. Podoconiosis has been included national program and masterplans. The inclusion of podoconiosis in the national strategic plan is important as NGOs and funders could be encouraged to fund and work on interventions that area part of the national strategic plans. | - Podoconiosis finally became a national priority in 2013 and was included in the National Master Plan for Neglected Tropical Diseases (NTDs). The Master Plan was developed in order to provide strategic direction for the implementation of the prevention, control, and elimination of podoconiosis and other NTDs (Ministry of Health, 2016). - The Ministry of Health developed integrated Lymphatic Filariasis and Podoconiosis Morbidity Management and Disability Prevention Guidelines (Ministry of Health 2016). These guidelines provide evidence based practical guidance on patient management and disability prevention. - In 2019 Ethiopia rolled out a strategy to support intervention against NTDs using the Water, Sanitation and Hygiene (WASH) program. The WASH program could play a very crucial role in controlling NTDs including podoconiosis since the prevalence of NTDs is highly associated with inadequate hygiene and sanitation conditions. # - The WASH program aims to increase access to [safe water](https://en.wikipedia.org/wiki/Water_supply), adequate sanitation and hygiene education (Ministry of Health, 2019). In order to strengthen coordination between relevant sectors and stakeholders, Ethiopia has collaborated with World Health Organization (WHO) to create a national WASH-NTD framework. Subsequently, a district level coordination toolkit for NTD and WASH sectors has been developed to guide implementation of health programs against NTDs. The toolkit provides practical guideline on how governmental sectors such as water, education, health and finance and NGOs shall collaborate at grassroot level (Ministry of Health, nd.). - The Government of Ethiopia has also developed a National Hygiene and Environment Health (HE) and Neglected Tropical Diseases (NTD) Message Guide to support NTD services. The guide is meant to communicate core messages regarding podoconiosis treatment and prevention methods with the intention of diffusing consistent messages across various communication channels (Ministry of Health, 2018). |
|  |  | Political will at regional level | The regional government demonstrated its commitment in setting up the NTD structures at zonal and woreda levels.  . | - We held a project launching workshop. At the launching we concluded a memorandum of understanding with key stakeholders in the region to facilitate collaboration and partnership for implementing the project (health bureau, finance, and economy, etc. (KII, staff of intervention implementing NGOs). - The NTD section has been established in the health sector structure at regional, zonal and district level to account for these neglected health problem.” (KII, NTD officer, Yilmana Densa District). |
|  |  | Political upheaval | The conflict in the northern parts of the country influenced the integration process in a sense that, the local structures had diverted their resources and attention towards the war and unable to sustain provision of treatment supplies and to patients. | - The civil war in the northern part of the country also influenced the intervention. The civil war diverted the attention of the government and community on security issues (KII, District NTD officer) - … this project wouldn’t have become successful without active involvement of the political elites of relevant government sectors and local structures.” (KII, Regional NTD team, podoconiosis coordinator) - Overall cooperation received form the regional health bureau to promote the integration process was highly encouraging.” (KII, IOCC staff) |
|  | **Legal context**  The legal domain encompasses the rules and regulation that aim to protect the populations’ rights. | Governments obligation. | **Macro level**  Governments obligation. The government has a legal obligation to provide adequate clothing to protect patients and at risk people from podoconiosis and acute attacks. | Ethiopia ratified the International Covenant on Economic, Social and Cultural Rights in 1993. (UN, nd). [UNTC](https://treaties.un.org/Pages/ViewDetails.aspx?src=IND&mtdsg_no=IV-3&chapter=4). United Nations Treaty Collection. The States Parties to the present Covenant recognize the right of everyone to an adequate standard of living for himself and his family, including adequate food, clothing and housing, and to the continuous improvement of living conditions. The States Parties will take appropriate steps to ensure the realization of this right, recognizing to this effect the essential importance of international co-operation based on free consent. |
|  | **Ethical context**  The ethical domain comprises reflections of morali­ty, which encompasses beliefs, standards of conduct and principles that guide the behavior of indivi­duals in their private and professional capacity and of institutions. It includes moral values at stake or in conflict, as well as those constructed by putting the intervention into use (EUnetHTA, 2010). | Lack of commitment among staff | Meso-Level  Staff at healthcare centers lacked motivation to engage themselves in podoconiosis treatment. | OCC provided training at the initial phase to the health staff from each clinic in Debre tabor town at zonal health office. However, after the training, the trained staff were not actively engaged. They were not interested to take on this task. We insisted the staff to take it seriously informing them that it is against the medical ethics and integrity of the profession (KII, NTD officer, Dera District) |
|  |  | Apathy to follow instructions | Meso-level:  Some health professionals were reported not to follow instruction given by the IOCC. | As per the previous research on willingness to pay for treatment, findings show promising results that substantial proportion of the households are willing to afford for treatment. These options could be taken as alternative to minimize expenses and sustain the services. However, most of the health professionals at Healthcare Centers did not mind about prioritizing patients based on their poverty level but provided materials on first come first served basis including for those who are well off and able to afford these materials. This created dependency among the well-off families and disappointment among the poorest and discouraged them to continue service (KII, staff of intervention implementors) |
| Implementation  Implementation is an actively planned and deliberately initiated effort with the intention to bring a given intervention into policy and practice within a particular setting. (Pfadenhauer et al. 2017). | **Implementation goal** | Improving quality of lives. | The aim of the project was to ensure and strengthen integration of the podo operation in the target health centres. | The aim of the project is to improve the lives of individuals affected by podoconiosis in severely and heavily affected districts in the Amhara region of Ethiopia (NaPAN and IOCC, 2020). |
|  | **Assumptions** | Mobilizing the community resources | The project activities can be successfully implemented by utilizing the community resources and actors. | - HEWs know the community even at the household level and hence they can easily identify podoconiosis cases to refer to health centers and provide patient follow-up. - Patient-led groups are living witnesses to the change they have seen as a result of the treatment they have received and hence they can easily influence other patients. - The patient-led groups/association structure could be considered as very sustainable and can be capitalized on for addressing and positively impacting podoconiosis patients and others in endemic communities. - Community leaders can access the community easily in their day-to-day rituals; and - Credible and carefully designed messages or advice they are passing onto the community are easily accepted (NaPAN & IOCC, 2020, pp 21) |
|  | **Implementation Strategy**  The implementation strategies comprise the methods (e.g. preferred and tailored activities,) and means used by the intervention implementing organization to ensure the adoption and sustainment of intervention (Pfadenhauer et al. 20017). | Integrated Approach | The NGOs utilized local healthcare centers in a bid to mainstream podoconiosis services into local structure. The project was informed by IOCC’s past intervention evaluations that suggested standalone or direct projects were less cost effective. In line with IOCC global, strategic plan, IOCC Ethiopia Made a paradigm shift from direct intervention to integration model. Contextualizing it with the National NTD master plan, the main assumption was to optimize treatment impacts among podo patients through building capacity of the local health structures. | “…this approach created advantages in sharing resources and experience, minimizing resource duplications and sharing knowledges among the NTD team. (KII, staff of intervention implementing organization)  “Implementation design of this project employed integration approach along the line with the national Master plan with the intention to link the services with the local health structures …it serves as a learning exercise for future intervention. (KII Regional NTD team leader) . |
|  | **Implementation Process**  This refers to the intervention process operationalization in the intervention implementing organization. This includes the planning of the implementation process, initial and full execution of activities, corrections, refinements and expansion of the intervention (May et al. 2009) | Cascaded training and health staff capacity building development | The intervention has familiarized health staff in local health structures with podoconiosis and enhanced their confidence to provide treatment and counseling to patients affected by podoconiosis. Some of the trained health professional provided trainings for health extension workers. | - I took part in the training conducted by the NGOs. The main topic covered all NTDs that are common in Ethiopia, but the focus was on podoconiosis, Lymphatic Filariasis and Hydrocele. The training was provided last year in 2021. Three staff form this health center, including me have been trained. I would say that the training has opened my eyes about podoconiosis. I had no information about this disease before the training. I have learned a lot from the training; gained better understanding about the causes and treatment. We have also been trained on how to provide counseling to patients. Trainers shared their rich experience on podoconiosis, and presentation were supported with visual aids, video, and group exercises (KII, Podo focal person Yilmana Densa District). - We have explained to the HEWs about the purpose of the intervention and its procedures and their specific role in the intervention. Their role was to mobilize and send patients to the health center. That was their only duty. - A trained focal person from Agita Health Center given us a half-day training on podoconiosis. He gave us orientation about the disease using training manuals (Health Extension Worker, Yilmana Densa District). |
|  |  | Distribution of treatment supplies |  | - Patients were provided with shoes, soap and washing tubs for treatment. I have seen in my eyes that patients showing up at the health center and receiving these treatments. However, I am not sure how many of them have received the support and how long the service has continued. There was such an initiative in our community (FGD with Women, Dera District). - Yes I have been enrolled. Health extension workers summoned all patients in the villages and informed us to go to the health center to take treatment. Accordingly, I went there three times, ones in every month last year. There were so many patients with me. On the first and second visit I was given soap and Vaseline oil and a plastic washing basin, and they washed my feet with soap. They informed us to wash our feet every day at home. On the third visit, I was provided with shoes and soap (IDI, affected male, age 18). |
|  |  | Treatment demonstrations | Along with introducing self-care and treatment procedure to patients, the feet washing demonstration sessions created opportunity to put across message to patients and to the community that the disease is not contagious. | - “I have seen in my eyes… Health staff washed their feet, touching them with their hands. It gave me the lesson that it is not transmittable with skin contact.” (FGD participant, male group, Dera Woreda). - The health staff were washing our feet and we were informed that when the service terminates, we should continue applying the treatment procedures at home (IDI, affected male, age 60). - During the intervention, the health staff informed us that this disease can be treatable by washing feet and wearing shoes. The health staff soaked our feet in water in a plastic bowl and showed us how to wash the feet (IDI, a 25 years old affected male) |
|  |  | Establishing Patient Association | Patients’ association have been established by in each district | The aim of the association was to establish our own institution to assist patients with economic and social support in the future. We were informed by the staff that the support from the health center may not last long (IDI, A56 years old affected female) |
|  |  | Risk communication (although intermittent) delivered at the health centers and religious settings. | Although minimal, messages disseminated at the health centers and spiritual places sparked the idea among some community members about the main cause and shoes wearing and foot hygiene as key prevention. Informants suggested extensive community level awareness to change attitude against patients. | - “Trained staff and health extension workers provided health education to the community about podoconiosis at the health center and in the churches.” (KII-06, NTD officer, Dera Woreda) |
|  | **Gaps and challenges in implementation strategy and assumptions** | Defaulting | A number of affected individuals have defaulted from the intervention activities | - They expect patients to come to healthcare centers to get the service. I couldn’t come to the health center because my village is about 3 hours far from the health center and can’t not come to the health center regularly for the service that was not helpful and effective ((IDI, a 25 years old affected male) - I went to the health station as we were told by the HEWs a new medicine has arrived. The health professionals showed us how to take care of our feet and provide us with soaps. I didn’t go in the second round. Why would I travel with my ill leg for three bars of soap? (A 45 years old affected man) |
|  |  | Staff turnover | Staff turnover at healthcare center. Trained health professionals quitted and that left a gap in implementing Lymphedema services. | - Staff turnover was another main challenge faced in the implementation process (KII, Intervention implementing organization) |
|  |  | Lack of community mobilization | There has been little attempt to mobilize the wider community in the project. | - The other component lacking in this project was a robust community awareness . A one time shot many not be helpful to bring sustainable change. A well-planned community campaigns involving religious and community leaders might be crucial to the achieve community-wide adherence to treatments and prevention practices (KII, staff of intervention implementing organization). - Community participation was low. It would have been better if we could strongly work with the local influential people in the community to change attitude of the people about shoes wearing and social stigma against patients. continuous awareness creation and training should be provided to the community leaders and volunteers in the community to bring a better change. The nature of this disease requires a lengthy interventions period of treatment, and it takes time so, continuous awareness should be given to patients and encourage role models who made changes through such interventions to share experiences to other patients( KII, NTD Officer at Dera District). |
|  |  | Lack of follow ups | The health staff didn’t make a sufficient attempt to follow adoption of treatment and other activities | - Podoconiosis intervention requires continuous effort as it take long time to cure the disease. I cannot say that our intervention for three months alone brought significant impact. I am telling you just from my observation. Formal evaluation has not been done. Even at the health center and woreda level, podoconiosis has been ignored in the regular monitoring and follow up unlike other health services (KII, podo focat person, Yilmana Densa District) |
|  |  | Overburdened and unmotivated staff | The staff at intervention delivery points were overburdened by other activities and were less motivated to implement services | - Not only the staff but also the woreda health office has given little attention towards this intervention. It was just like a onetime shot, and no one was concerned about the follow up and continuation of the intervention. Staff seem to be faded up and relinquished their responsibility. The other challenge was workload that I have in my office. I have multiple roles to play in this health center. For instance, I’m the coordinator of the health extension program, I also coordinate the youth reproductive health program, and participate in recurrent national and regional health campaigns and vaccination programs alongside with my regular duty as clinical nurse treating outpatients every day. Podoconiosis is additional load to my job although I am highly concerned about the problem (KII, Podo focal person at Yilman Densa District). - Health professional sometime were overburdened with recurrent health promotion campaigns in other health programs (KII, Staff of Intervention Implementing Organization). - During the review meeting with health centers, all the centers have reported that they have done the cascading, but with some challenges specially complaints from health extension workers for incentives have been made. HEWs are given incentive when they administered drugs. There is no incentive for them to work on podo. As a result, they are less motivated to focus on podo (KII, Staff of Intervention implementing organization) |
|  |  | Some patients felt that the intervention can not meet their needs |  | - In my opinion, this treatment it is not sufficient. I wish if higher lever government officials could have visited us to find the lasting solution for this problem. This treatment only helped to minimize the swelling. It does not help patients who have hereditary predispositions like in our family. The health center staff provided us advice only washing feet could make a difference. I don’t think this is true. They do not provide us medicines to treat the disease. I don’t think washing feet alone is the best treatment. We need a better medication to cure the disease. Washing the feet and taking care of the feet with soap and massaging it with oil requires frequent and consistent attention every day (IDI, a father of a female patient, age 63). - Frankly speaking, the benefit was not that much promising. There is only a slight change on the swelling. Of course, my feet have become smooth and free of wounds but the swelling still bigger and sometimes I experience acute attacks (IDI, a 18 years old affected male). - Patients often come to the center with the expectation to receive material support and disregarding the education session and treatment procedures. |
|  |  | Non-functionality of local structures | The local structures that the NGOs had planned to utilize to execute the project activities were not functional. | I have doubt that Health Development Army leaders may have not been reached through this process. I have also a doubt that HDAs and the structure itself was not as active as it should be. Had they been well trained, they could have mobilized more patients and facilitated follow ups at the household level. These is one of the major gaps we realized form our supervision reports. |
|  |  | Budget constraints | Budget constraints had affected the outreach and execution of the intervention | - Budget shortage for the NTD section. Although the structure is there at zonal and district level budget has not been earmarked to this structure. The project itself has been implemented with limited amount of budget. Our plan was to reach 7000 patients. However due to limited resource we were not able to reach all the patients. Specially shoe distribution was affected by the shortage of budgets. My main concern is the resource constraint. After April 2023 we will hand over the project to the health Bureau. Unless the bureau allocates budget to this initiative the continuity of the project will face the risk of termination. I would hope that the regional health bureau will take it seriously as per our memorandum of understanding (KII, Staff of intervention Implementing organization). - Based on that we have made supervisions and observed that podo focal staff in most of the health centers were actively engaged in providing the training to HEWs. We practically observed this in some health centers. In fact, we were not able to see the situation in each patient household due to budget limitations. We realized that there were variations in achievement among health centers (KII, staff of intervention implementing organization). - IOCC had planned to cover 20% of the cost for shoes and washing basin to be delivered to the poorest of the poor patients and the remaining will be contributed by the health center. (KII, staff of NGO). |
|  |  | Dependency syndrome | Free handout and inconsistent distribution of shoes and supplies created dependency and complaints among patients. | - It would have been good if all the patients have been given appropriate shoes and medical support to help them get instant cure (An affected male, age 25). - I know that some patients went to the health center, and they have received leather-made shoes washing basin and soap for treatment. Although I lately went to the center this year and asked the health staff to register me for support, they informed me that it was too late, and no service was provided at that time. I was wondering why they did not call me for such important service. I suspect health staff at the health center might have abused the budget allocated by the government for our support and refused to inform us (An affected female, age 38). |
|  |  | Non-functional patient association. | The patient association were barely functional and affected individuals see little relevance in them. | - Before the association stood on its feet, most patients started complaining about its lack of support. Although members contributed 5 Ethiopian Birr (currency) each month, they don’t see any benefit. They demanded instant support and some patients withdrew from membership taking their money back. This situation entailed discouragement among members, and I would say that currently this association is almost not existent, not functional. No one is attending the monthly meeting. Even the staff were not committed to push it consistently (IDI, affected woman, age 56) - We were informed about the importance of establish patient-led associations by IOCC. We coordinated the establishment of the association in our district. The idea was to build the economic capacity of the patients, so that hey would become self-sustained in treatment and economic life. The association didn’t progress as we expected. Members contribute 10 Ethiopian Birr per monthly. There was a high expectation of patients for a quick return from the savings leading frustrations among members and lack of trust on the association. I suspect that the staff at the *Arb gebeya* health center might have provided them an over ambitious pan of the association which didn’t live up to the patients’ expectation (KII, NTD officer Dera) |
|  |  | COVID-19 | The COVID-19 pandemic has affected the implementation of the intervention activities | - COVID-19 has affected the progress of our implementation. The outbreak of the pandemic was announced soon after we started the project. the physical distancing and containments strategies have affected mobility of the staff and all essential services at the health centers were challenged. Therefore, the joint monitoring and review meetings were not conducted due to these circumstances (KII, staff of regional health bureau). - COVID-19 was one of the major challenges we faced. We managed to provide the training for 183 trainees underneath the COVID-19 pandemic challenges where public gathering was restricted. We did this limiting the number of trainees at a time and splitting them into a small group and deploying more trainers (KII, staff of Intervention Implementing Organization) |
|  |  | Fear of Stigma |  | - I went to the health station first and I had little reason to go there for the second time….I would rather stay at home. People will stare at your feet and make you feel uncomfortable…What is the point of going to the health station? If you stay at your home, no one hurts your feeling (a 32 years old affected woman). - Most patients refrain form coming to the health centre for other health services, simply because they were ashamed of their condition. Only a few patients used to come at the health center. They don’t want to come just to avoid the reaction of people towards their feet and the bad odour that keeps people in distance from them (KII, podo focal person at Yilman Densa District). - Patients don’t want to show up on meetings organized to educate the community about podoconiosis for fear of stigma. We would rather call a separate meeting for patients in the event when we want to educate them about it or go for a home-based education. Even when we provide education in the religious institutions mixed with healthy individuals, they become the focus of attention of others and that create humiliation among patients (KII, HEWs at Yilmana Densa District) |
|  | **Implementation Agents**  Implementation agents comprise all individuals and organizations engaged with (i) deciding to implement a given intervention (e.g., funders, administrators), (ii) implementing the intervention (e.g., providers,), (iii) being the target or otherwise affected by an intervention (individuals affected by podoconiosis) (Pfadenhauer et al. 2017). | Funders | Project funder/donor agency – IZUMI Foundation  The Amhara Regional Government signatories (Health Burau, Economic planning and Finance, Bureau, Social Afairs Bureau and Education Bureau), IOCC head quarter. | “IZUMI foundation our longstanding funder to IOCC’s podoconiosis project since 2016.” (KII, IOCC staff) |
|  |  | Implementing actors | IOCC Ethiopia and NaPAN jointly implemented the project | The next Step project was implemented by IOCC and NaPPAN where IOCC was responsible for implementing the intervention and whereas NaPPAN was responsible for supervision and evaluation. “(KII, Regional NTD staff) |
|  |  | Intervention targets | The intervention targets individuals affected by podoconiosis, local community and health professionals. | - We had a plan to train health professionals and provide treatment for 7000 patients. We further provided health education at healthcare centers for the community (KII, staff of Intervention implementing organization) |
|  |  | Collaboration partnership and participation | Collaboration and commitment of government structures have been helpful implement the project.  Community participation is minimal. Bottom-up participation should be employed to maximize community mobilization and participation in the project. | - The Amhara Regional Health Bureau welcomed us when we inquired to recruit seconded staff and operate through integration approach along with the NTD team in the regional health office. As the NTD team is responsible to all types of the NTD related interventions, this approach created advantages in sharing resources and experience, minimizing resources duplications and sharing knowledges among the NTD team (KII, staff of intervention implementing organization). - Community participation was low. It would have been better if we could strongly work with the local influential people in the community to change attitude of the people”. (KII, NTD officer, Dera Woreda Health Office) |
|  | **Implementation outcomes**  Outcome is the result of the implementation effort. In this study, we focused on the fidelity, acceptability and sustainability of the intervention. Fidelity refers to what extent the implementation strategies are being implemented as per the plan (Gearing et al. 2011). In addition, we will study to what extent the intervention is being accepted by the intended beneficiaries and what changes are likely remain after the intervention. | **Acceptability** | **Knowledge about the purpose of the intervention**  Most of the patients in the community were well informed about the purpose of intervention and the types of services provided by the health center.  On contrary, the general community (most of the unaffected community members) seem to have limited information about the intervention or its purpose. | - Most patients have received education and attended the demonstration session at the health center...(KII, HEW Yilmana Densa Distric). - I have benefited from these interventions in a sense that my knowledge about the disease has been improved (IDI, affected, male age 54) - Patients hold better understanding than other people in the community…they have received information form the health center. I think healthy people have wrong understanding about it. (KII, Local Leader,Yilmana Densa district) |
|  |  |  | **Perception towards the significances of the intervention.**  Most respondents alluded intervention has introduced accessibility of treatment to podoconosis patients at the health centers. This initiative also created the impression among most patients and non-patients that podoconiosis disease can be treatable | In the past, we thought that this disease could not curable. Now that we have seen some indications that convinced us to believe the disease could be treatable and curable. Some patients have been recovered from it. (FGD participant, Women group, Yilmana Densa District) |
|  |  |  | **Demand creation and acceptance to treatment**  High enrollment /show up of patients and a considerable number graduating through the three months follow up period explains treatment acceptance among most patients. | - We did the mobilization door to door, at the church and in the schools to inform households to send podo patients to the clinic to receive the intervention. Accordingly, there was high turnout out of patients at the beginning.” (KII Podo focal staff ). - I went there three times, ones in every month last year. There were so many patients with me.” (IDI, young male patient, age 18.) |
|  |  | **Fidelity.**  Fidelity refers to what extent the implementation strategies are being implemented as per the plan (Gearing et al. 2011). | As per the plan, the NGOs were able to train local health professionals and commenced the integration of podoconiosis within existing health facilities.  **Gaps:** Intervention was designed to cascade the treatment through the existing health structures. However, it mainly concentrated at the health center setting and not well trickled down to lower structure particularly in engaging Health Development army (HDA) and health extensions programs at the health posts | - We found that most of the health centers were doing well … Cascading trainings have been implemented in most of the health centers. In many of the health centers some patients show up regularly for follow up visits. High attendance means there is good linkage with the health extension workers. The review meeting informed us that there was a change in the reduction of acute attack among patients. (KII, staff of intervention implementing organization) - The intervention was not functional at the health post level. It was limited to the health center. We have limited follow up of patients after the three rounds of intervention at the health center (KII, NTD Officer, YD woreda Health Office). - We have not received any training on podoconiosis. It was given only to the health staff at the health center, not for health extension workers. We were given just a quick briefing or orientation by the health staff and observed the demonstrations performed by the health staff on how to wash and bandage the affected part of the feet. That was not sufficient for us to understand the condition in detail (KII, HEWs at Dera District) |
|  |  | **Change on stigma**: Reported Intervention impacts that likely contribute to changes on stigma**.** | Reduction of acute attack improved patients’ time to engage in economic activities. This resulted in turning around the prevailed sense of economic inferiority among patients. | - There is a stark difference compared to the past (before the intervention). A significant improvement has seen among many patients with regards to reduction in the magnitude of occurrence of acute attack and the offensive odor (KII, NTD Officer at Dera District Health Office). |
|  |  |  | The treatment has improved physical wellbeing among those adhering to the procedures in a sense that their footwear and feet hygiene practice boosted their self-worth and participation in social events and overcoming self-stigma. | - I have seen improvement among those who have received shoes and soap. Their feet became clean and neat. Unlike the past, their feet have no bad odor.” (IDI, affected male, age 40). |
|  |  |  | The intervention has introduced belief about treatability of podoconiosis. This has been noted by informants as important pathway to bring change on self-stigma of patients and public misconceptions. | - This intervention also sparked some light on patients understanding about the treatment procedures…also contributed for reduction of self-stigma and self-isolation of patients (KII-, NTD Officer, Yilmana Densa District) |
|  |  | **Barriers to stigma reduction.** | Prevailing misconceptions and lack of clear awareness about the cause of podoconiosis. (Stepping on goat’s blood, sharing washing tools, contacts with patients, etc.) | - Toilets harbor hook worms. Walking bare feet in these places put people at high risk for exposure to hookworms that eventually leads to podoconiosis” (KII, HEW, Dera District) - Hereditary swellings can’t be cured. It is the will of God (IDI, Affected male, age 40) - Mostly my clients report the main cause as washing the feet by the river side during hot seasons.” (KII, male traditional healer in District) |
|  |  |  | Lack of adherence to treatment: Expectation of instant cure from the treatment, high dependency syndrome and misunderstandings about the intervention accounted for many patients to drop out the treatment. The condition reinforced concerns over beliefs about curability of the disease that leverages the stigma reduction. | - I have given up as soon as the soap and ointments are run out...I cannot afford buying soaps leave alone these types of shoes. I am still waiting for shoe support” (IDI, Affected male age 64) |
|  |  |  | Economic challenge encountered by most patients influenced their access to treatment supplies and thwarted motivation to self-care. The economic burden also intensified inferiority feeling among patients manifested in social life. | - I didn’t hear about the intervention. I didn’t receive any supplies….[Why don’t you buy soap and wash your feet?]…Surely you jest ! Do you think I can buy soap every time to wash my feet?! |
|  |  | Sustainability | It was assumed that mainstreaming podoconiosis in the NTD structures and health centers reinforce sustainability of self-care treatment and ultimately improve the physical, social, and economic wellbeing of patients including stigma reduction.  It was assumed that patients will continue practicing the footcare instructions they were given during the intervention. Some patients lost trust on the treatment intervention due to lack of perseverance to the lengthy process required to see improvement. Such expectations to quick recovery have discouraged patients and to hold perception that podoconiosis is untreatable. | - The aim of the project was to ensure and strengthen integration of the podo operation in the target health centers” (KII, IOCC staff) - Integration was taken as a means for sustainability as it provides opportunity for creating access and open doors services for patients, which was also believed to eventually contribute to stigma reduction (KII, District NTD officer) - I tried to wash my feet and bandage it for a few weeks, it was useless… I thought it was curable, but I have not seen any change”. (IDI, Female patient, age 50) - The health professional told me that it is curable, but I don’t believe it is true. There is no promising improvement in my condition since I have started the treatments. I gave up hope all together. So, I don’t follow the instruction anymore (IDI, male, age 18) |
| **Setting**  Setting refers to the specific physical and organizational environment in which the intervention is put into practice and interacts with context and implementation (Pfadenbaurer et al. 2027) | **The physical environment** | Intervention delivery point | The intervention/ treatment was provided to patients at the health centers clustered to make accessible to all kebeles in the districts, by podo focal staff, accountable to the District NTD office. While participants show up at the health centers for demonstrations and follow up visits, they were practicing the treatment in their homes.  Intervention was designed to cascade the treatment through the existing health structures. However, it mainly concentrated at the health center setting and not well trickled down to lower structure particularly in engaging Health .Development army (HDA) and health extensions programs at the health posts. | “The Arb Gebeya Health Center serves around 40,000 residents in 5 kebeles in Dera woreda. Each kebele has one health post accountable to the health center. The health center consists of 61 employees, of which 13 are Health Extension Workers, 1 physician (Medical Doctor), 5 health officers, 5 midwifes, 2 laboratory technicians, 8 clinical nurses, 3 pharmacists, 4 PSG nurses. The rest are administrative staff.” (KII, Manager, Health Professional, Dera District).  It is a normal day to day practice in the health center to provide health education in the morning in the waiting area before the service starts). The waiting area is situated in front of the main gate of the health center with an approximate occupancy of 20-30 persons in ventilated open and high roofed verandah. A television set is placed in the waiting area from which patients enjoy getting local and national information broadcasted by the mainstream media including health related information. For reasons unknown, the television was not switched on during the time of our arrival, rather it was locked in a television stand box. A big poster has been put up on the wall of the waiting area carrying messages about the rights and obligations of patients. The waiting area has limited seats or benches installed in a u-shaped corner. On each building, in addition to the waiting area, various posters and banners have been put up conveying messages such as the type of services being provided at the health center, and awareness raising messages about various diseases. However, there was no poster or written text messages displayed about podoconiosis at any corner of the building (Observation at health center, Yilmana Densa District)  “…the intervention was not functional at the health post level. It was limited to the health center. We have limited follow up of patients after the three rounds of intervention at the health center (KII, NTD Officer, Yilmana Densa Health Office) |
|  |  | Implementors’ office | IOCC and NaPAN’s offices are located in the capital city of Ethiopia-Addis Ababa. Although NaPAN has a cramped office as compared to IOCC, the offices in the two organizations have sufficient spaces and equipment to smoothly run projects. | The IOCC office is located in the eastern part of the capital Addis Ababa, near to the Civil Service College, in the Cheshire Service Ethiopia’s building, on the 4th floor. The largest section of the floor has been occupied by IOCC and a couple rooms are owned by other private organizations sharing the same hallway with IOCC. All its office rooms are situated on both sides of the hallway. As we get into the 4th floor using an elevator or the stairs, the is a narrow corridor in front of which we find partitioned rooms, of which, the first one is the office for the IOCC’s country representative. To the left side, along the hallway there are staff toilet rooms shared with other organizations in the same floor. Next to it, there is a closed door with a transparent logo of IOCC printed on it; which is the man entrance to all other IOCC staff rooms. In short distance from the main iterance, we find the office for the podoconiosis program manager on the left side of the corridor. In front of it, there is a multipurpose room which serves as an office for support staff (drivers, and office attendants), a printing room and a teatime area where employees come together during break hours to have coffee or lunch and meet and interact along the way.  Next to the podoconiosis program manager’s room, we find the office for Administration and Finance Head. Next to it is a meeting room furnished with a traditional seats and tables with occupancy of about 15 people. Adjacent to the meeting room, to the right side of it, there are three office rooms in a raw for the Monitoring and Evaluation Officer, Finance officer and Livelihood program manager. Except the room for administrative assistant staff, all other rooms are occupied by one staff (one desk per one room). It is arranged in such a way that each staff has their own separate rooms and yet, unless for exceptional cases, all the rooms doors are left open during working hours to encourage open door working environment. Most staff had lunch in the office which his also a good opportunity for them to interact and keeps them closely attached and enhance teamwork (Observation at IOCC precinct). |
|  | **Organizational culture** | Vision, mission and values | Bureaucratized structure, clear missions, and visions as well as the reputation of IOCC and NaPAN could be an asset to register more success. | Following the new Ethiopian Organization of Civil Societies Proclamation in 2019, NaPAN developed its second strategic plan in consultation with stakeholders in 2020. The strategic plan shows that the organization’s vision is to see a podoconiosis-free Ethiopia. The mission of the organization reads as “NaPAN strives to coordinate and standardize efforts for the elimination of podoconiosis, through building the capacity of members and other stakeholders and supporting research for evidence-based interventions”. The organization plans to achieve its mission through the following strategies: using holistic and integrated programming; expanding or increasing consortium members; enhancing partnerships; fostering participatory approaches; using a community-based approach; building, strengthening and maintaining networks, creating alliances and forums/with national and international partners; strengthening evidence-based program development & management, conducting research, developing capacity for members; and influencing and facilitating dialogue on issues of podoconiosis/NTDs. The core values of NaPaN that are supposed to dictate the behavior of its staff are transparency, accountability, loyalty, integrity, professionalism, partnership, participation, quality service, impartiality, and voluntarism. These values are expected to reflect the importance the staff attach to the organization beyond providing a livelihood (Observation and document review at NaPAN) |
|  |  | Organizational structure staff profile | It appears that the staff and management at the two organizations have good collaboration and shared view on the project with clear information communicated among each other’s. There is a clear management structure role division and accountability to the project. Staff and management are highly enthusiastic about the project and are eager to see the outcome of this assessment, learn from the impact of their interventions. | During the observation period, IOCC had 16 employees (8 males and 8 females) running the day-to-day activities of the organization while NaPAN had four staff. Among the 16 staff of IOCC, 9 were program staff and 7 administration/support staff. Twelve of them have their duty station in the head office in Addis Ababa; while four employees (podoconiosis project coordinator, service field officer, project nurse, and a driver) are seconded staff stationed at the Amhara Regional State Health Bureau. The organization is spearheaded by a country representative (a female Ethiopian professional). Staff functions are set into two wings i.e. the program and administrative sections. The program section encompasses four distinct but complementary programs; each are headed by the program managers. These include: the podoconiosis program, livelihoods program, nutrition program and monitoring and evaluation (M and E) program. The podoconiosis program manager leads projects related to podoconiosis with the project coordinator, project officer and one nurse who are serving as seconded staff in Amhara Regional state Health Bureau. The M and E officer responsible follows up and monitors progress of each program being run by IOCC. All program heads are independently accountable to the country representative. The program support section is led by the administration and finance head that includes one finance officer, two drivers and three office assistants. The admin head is also accountable to the country representative (Observation at IOCC) |
|  |  | Monitoring and evaluation | Monitoring and evaluation activities have been given due attention by the organizations to provide feedback on the progress, outcomes and gauge the progress of the project | Project plans, supervision reports and project monitoring and evaluation reports are duly recorded and filed in both organizations. The project activities were being monitored using the supervision guide and deploying a supervision team quarterly to the project sites to observe progress of activities, achievements, progresses, challenges encountered and feedback. After the first year of the implementation phase, IOCC’s role in the project was limited. However, it has continued participating in the joint monitoring /supervision activities in collaboration with National Podoconiosis Action Network (NaPAN). |
|  | **Activities and innovativeness** | Measuring success using outreach and coverage | The outreach approach is on the major modus operandi. Admirably, the evaluated project was able to reach people affected by podoconiosis who never had access to health services before.  There is more than outreach and coverage about intervention against disease like podoconiosis. Although IOCC and NaPAN as organizations seem, to some extent, to have learned this important lesson, the staff still seem to measure their success on coverage. Overemphasis on technical aspects of the intervention could also affect the staff’s inability to come up with innovative and contextualized intervention. International donors inclined interest towards conditions that are suitable to drug therapy has affected the organizations and poses a great challenge in efforts to control podoconiosis | An admin head of NaPAN and I were discussing funds. believed that NGOs working on podoconiosis usually lack funds to design and run programs to meet the needs of patients. He felt that this is major reasons the project is only targeting 7000 patients. Of which only 700 of them would receive shoes. He further commented that it is pity that they can’t procure treatment supplies for every patient. What is important for the staff? Addressing the needs of 7000 patients can’t be undermined; provided the project address the needs of the patients. But why did the admin head consider the number to be small? What is his benchmark (Observation report at NaPAN).  There seems to be a tendency to over rely on supervision reports to measure the success of the intervention. As the supervision evaluation form emphasizes on quantifying and measuring individual level impacts, it could result in losing sight of the organizations’ goal to improve the lives of patient and reduce health related stigma (Observation report at IOCC and NaPAN) |
|  |  | Lack of Funds | Scant funding for drugless diseases has challenged the NGOs. The staff in both organizations equivocally believed that securing funds for podoconiosis has become increasingly difficult. A number of interventions against NTDs, particularly podoconiosis have been implemented despite little interest from international donors. Staff perceived that | Funders are less motivated to fund non-life threating and drugless diseases that are not amenable to advertise manufactured drugs (KII staff of intervention implementing organization) |
|  |  | Coping shortage of funding | To deal with shortage of funding, the NGOs are reinventing themselves by including other diseases that are suitable to MDA in their intervention programs | We have included Lymphatic Filariasis in our program. This increases funding opportunities (KII, staff of intervention implementing organization) |

1. [↑](#footnote-ref-1)
2. [↑](#footnote-ref-2)
